# Formal synthesis of (−)-agelastatin A: an iron(II)-mediated cyclization strategy

**DOI:** 10.3762/bjoc.9.99

**Published:** 2013-05-03

**Authors:** Daisuke Shigeoka, Takuma Kamon, Takehiko Yoshimitsu

**Affiliations:** 1 Graduate School of Pharmaceutical Sciences, Osaka University, 1-6 Yamadaoka, Suita, Osaka 565-0871, Japan

**Keywords:** agelastatin, aminohalogenation, iron(II), free radical, natural product synthesis

## Abstract

An iron(II)-mediated aminohalogenation of a cyclopentenyl *N*-tosyloxycarbamate provided new access to the key intermediate for the synthesis of (−)-agelastatin A (AA, **1**), a potent antiproliferative alkaloid. The present synthetic endeavour offered an insight into the mechanism underlying the iron(II)-mediated aminohalogenation of *N*-tosyloxycarbamate, in which the radical properties of the N–iron intermediates in the redox states were operative.

## Introduction

**Scheme 1:**
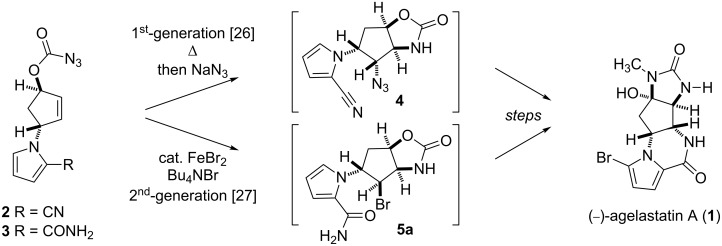
Our first- [26] and second-generation [27] approaches to (−)-agelastatin A (**1**).

Marine organisms often produce bioactive substances that potentially serve as attractive resources for drug discovery. (−)-Agelastatin A (AA, **1**), a cytotoxic alkaloid isolated from marine sponges *Agelas dendromorpha* and *Cymbastela* sp., is one such substance, which has drawn considerable attention due to its potential applicability in the development of anticancer agents [1–5]. The intriguing biological activity of **1** has stimulated interest in developing various chemical accesses to the natural product [6–25]. Our previous synthetic endeavours have established two approaches to **1**, in which cyclopentenyl azidoformates **2** and **3** were utilized as the pivotal intermediates (Scheme 1).

**Scheme 2:**
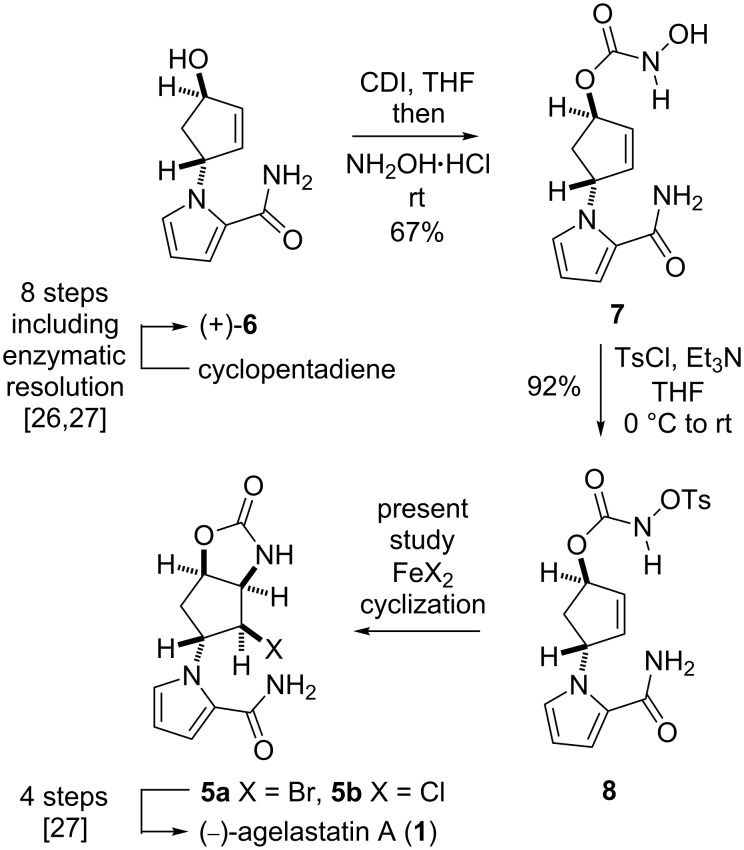
The present iron(II)-mediated aminohalogenation of *N*-tosyloxycarbamate **8** providing key intermediates **5a**/**5b** for (−)-agelastatin A synthesis.

The first-generation strategy employed a stereoselective thermal aziridination of azidoformate **2** and a subsequent aziridine-opening reaction to establish the vicinal *trans* nitrogen motif **4** [26]. The second-generation strategy involved the radical aminobromination of azidoformate **3** followed by lactamization of the resultant bromide **5a** to furnish a tetracyclic compound (structure not shown), which was transformed into the natural product [27]. In the present study, we disclose a new approach to the key intermediate for AA synthesis in which *N*-tosyloxycarbamate **8**, a nonhazardous azidoformate surrogate, is transformed into aminohalogenated compounds **5a** and **5b** by FeBr_2_/Bu_4_NBr [28–29], FeCl_2_/Bu_4_NCl, or FeCl_2_/TMSCl [30–35] (Scheme 2). Moreover, a plausible mechanism of the present iron(II)-mediated aminohalogenation, which is inferred from the unique reactivity of *N*-tosyloxycarbamate **8** with the reagents, is discussed.

## Results and Discussion

*N*-Tosyloxycarbamate **8** was prepared from alcohol **6**, which was obtained by a previously reported protocol (Scheme 2) [26–27]. Alcohol **6** was first treated with CDI (*N*,*N*’-carbonyldiimidazole) and then with hydroxylamine hydrochloric acid salt to afford *N*-hydroxycarbamate **7** in 67% yield [36]. Thereafter, *N*-hydroxycarbamate **7** was reacted with TsCl and triethylamine in THF to furnish *N*-tosyloxycarbamate **8** in 92% yield. With this carbamate **8**, we examined the iron(II)-mediated cyclization under various conditions (Table 1).

**Table 1:** Aminohalogenation of *N*-tosyloxycarbamate **8** by iron(II) catalysis.

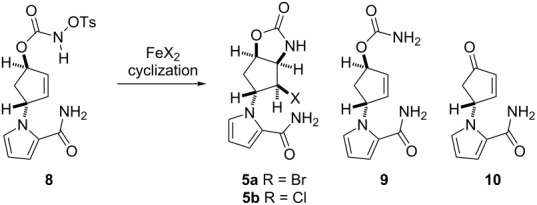		

entry	conditions^a^	products (%)

1	FeBr_2_ (0.5 equiv), Bu_4_NBr (1.5 equiv), EtOH, rt, 1.75 h	**5a** (13), **9** (39), **10** (30)
2	FeCl_2_ (0.5 equiv), Bu_4_NCl (1.2 equiv), EtOH, rt, 0.75 h	**5b** (39), **9** (20), **10** (19)
3	FeBr_2_ (0.5 equiv), Bu_4_NBr (1.2 equiv), *t*-BuOH, rt, 0.5 h	**5a** (38), **9** (22), **10** (19)
4	FeCl_2_ (0.5 equiv), Bu_4_NCl (1.2 equiv), *t*-BuOH, rt, 2.5 h	**5b** (48), **9** (9), **10** (9)
5	FeBr_2_ (0.2 equiv), Bu_4_NBr (1.2 equiv), *t*-BuOH, rt, 3.3 h	**5a** (25)^b^, **9** (16), **10** (5)
6	FeCl_2_ (0.2 equiv), Bu_4_NCl (1.2 equiv), *t*-BuOH, rt, 3.3 h	**5b** (31)^c^, **9** (9), **10** (14)
7	FeCl_2_ (0.5 equiv), TMSCl (1.5 equiv), EtOH, 0 °C to rt, 16 h	**5b** (29), **9** (12)^d^

^a^All reactions were conducted using 20 mg of substrate **8**. ^b^22% of **8** was recovered. ^c^35% of **8** was recovered. ^d^Compounds **11** (16%) and **12** (14%) were obtained.

**Scheme 3:**
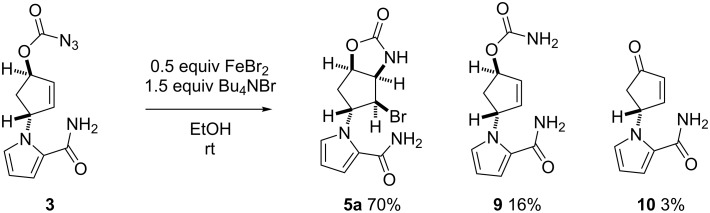
Aminohalogenation of azidoformate **3** (2 g scale) under FeBr_2_/Bu_4_NBr conditions.

**Figure 1:**
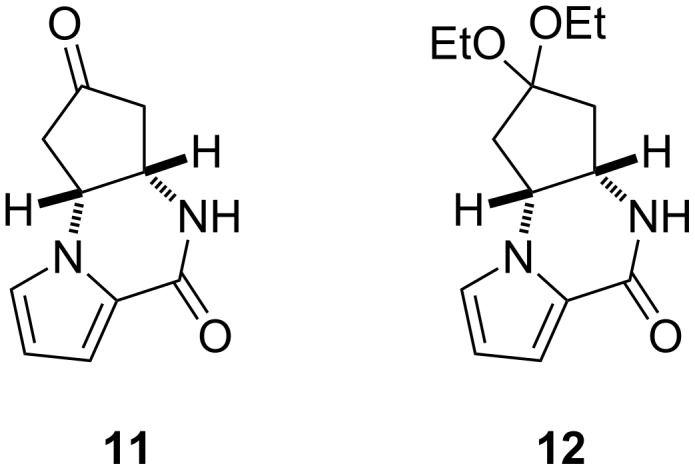
Byproducts formed by aminohalogenation of *N*-tosyloxycarbamate **8** with FeCl_2_/TMSCl in EtOH (see Table 1; entry 7).

The application of FeBr_2_ (0.5 equiv)/Bu_4_NBr (1.5 equiv) in EtOH effected cyclization, but the yield of **5a** was poor due to the concomitant formation of carbamate **9** (39%) and enone **10** (30%) (Table 1, entry 1). This was in marked contrast to the observation that the same reagent system, i.e., FeBr_2_ (0.5 equiv)/Bu_4_NBr (1.2 equiv), allowed the efficient conversion of azidoformate **3** (2 g scale) in EtOH to afford **5a** in 70% yield (Scheme 3). The distinct yields of the cyclized materials obtained from *N*-tosyloxycarbamate **8** and azidoformate **3** suggested the unique reactivity of each substrate towards the iron(II) halide (see below). An aminochlorination reagent system, i.e., FeCl_2_ (0.5 equiv)/Bu_4_NCl (1.2 equiv) in EtOH, in turn, furnished the corresponding chloride **5b** in 39% yield as the major product (Table 1, entry 2). Our recent studies on the iron(II)-mediated aminobromination reactions of structurally simple *N*-tosyloxycarbamates with FeBr_2_/Bu_4_NBr revealed significant solvent effects on the product yields [28]. This was also the case in the present study: FeBr_2_ (0.5 equiv)/Bu_4_NBr (1.2 equiv) in *t*-BuOH successfully improved the yield of **5a** relative to the reaction in EtOH (Table 1, entry 3). FeCl_2_ (0.5 equiv)/Bu_4_NCl (1.2 equiv) in *t*-BuOH culminated in the highest yield of **5b** among the examined conditions (Table 1, entry 4) However, a reduction of FeX_2_ loading even in *t*-BuOH led to erosion of the yields of halides **5a** and **5b** with recovery of the substrate (Table 1, entries 5 and 6). With the FeCl_2_/TMSCl reagent system [30–35], chloride **5b** was accessible from *N*-tosyloxycarbamate **8** in 29% yield, along with **9** in 12% yield (Table 1, entry 7). In this particular case, cyclopentanone derivative **11** (16%) and diethyl ketal **12** (14%) were produced as well (Figure 1). An additional experiment to elucidate the origin of their formation provided evidence that these byproducts were generated by the intramolecular cyclization of enone **10** with TMSCl in EtOH, suggesting that the FeCl_2_/TMSCl system also gave enone **10** in ca. 30% yield [37].

**Scheme 4:**
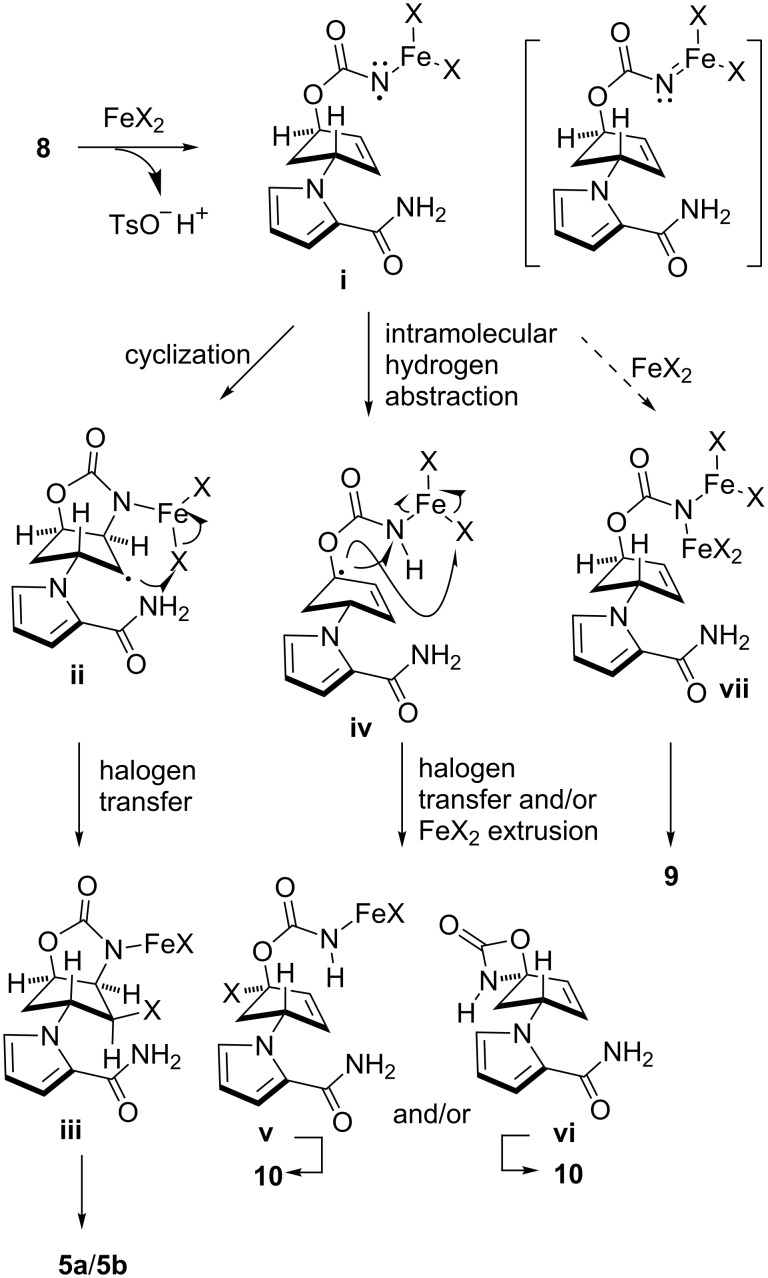
Plausible reaction pathways in the aminohalogenation of *N*-tosyloxycarbamate **8** with FeX_2_/Bu_4_NX.

The present study on the aminohalogenation reaction of carbamate **8** has inspired mechanistic insights that deserve discussion (Scheme 4). We hypothesize that cyclized material **5a**/**5b**, reduced material **9**, and enone **10** are generated from an N–iron complex (**i**) that has free-radical character, as previously proposed in the catalytic cyclization of azidoformates [30,38–40]. The contrasting yields obtained from *N*-tosyloxycarbamate **8** and azidoformate **3** under FeBr_2_/Bu_4_NBr in EtOH conditions (see Table 1, entry 1 versus Scheme 3) likely originate from the distinct chemical property of the N–iron species (**i**) generated from each substrate. The possible coordination of tosylate anion to the N–iron after the N–O bond cleavage with FeX_2_ may have affected the electronic and steric characters of intermediate (**i**), leading to retardation of the subsequent cyclization. Because of the low cyclization rate, the production of reduced carbamate **9** and enone **10** became pronounced. This is consistent with the observation that the relatively efficient production of cyclized material **5a** was observed for azidoformate **3**, where N–iron intermediate (**i**) was free from such interactions. One of the other characteristics found in the present transformations was the incomplete consumption of substrate **8** by lowering FeBr_2_/Bu_4_NBr loading (e.g., Table 1, entry 5), which, in turn, enabled the efficient conversion of structurally simple *N*-tosyloxycarbamates into the corresponding cyclic aminobromides [28]. This poor conversion under conditions of less FeX_2_/Bu_4_NX loading may be attributable to the decrease of the concentration of reactive FeX_2_ through capture with the polar amide functionality of **8**.

**Scheme 5:**
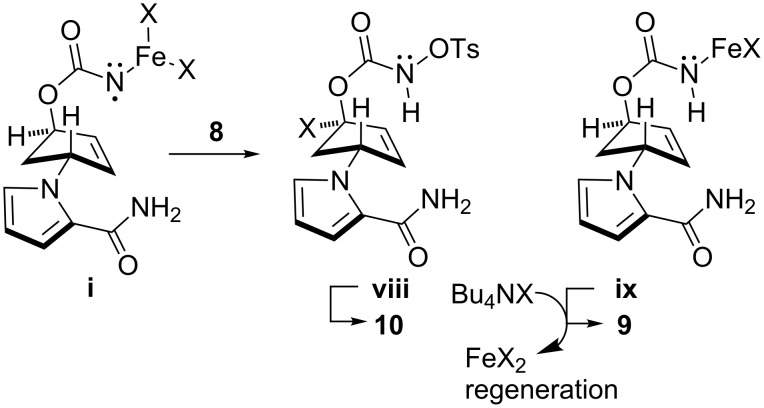
Plausible reaction pathway to produce compounds **9** and **10**.

It is speculated that product **9** may be produced by trapping N–iron complex (**i**) with another FeX_2_ (**i**→**vii→9**), whereas enone **10** is likely to be generated via intramolecular allylic hydrogen abstraction followed by halogen transfer to regenerate iron(II) species (**i**→**iv**→**v**→**10**) and/or by directly releasing FeX_2_ (**i**→**iv**→**vi**→**10**) [41]. However, it is worth discussing the process for yielding **9**, which theoretically generates two equivalents of iron(III) species per one equivalent of **vii**. Given the observation that FeCl_3_/Bu_4_NCl gave none of the products shown in Table 1, an iron(III) species possibly generated via the halogen exchange of **vii** with Bu_4_NX, if any, no longer has catalytic activity and thus the catalytic cycle is terminated. Therefore, active FeX_2_ species should somehow be regenerated to maintain the catalysis. One possible pathway that may account for the production of carbamate **9** through the regeneration of FeX_2_ species is the intermolecular hydrogen abstraction from substrate **8** by N–iron species (**i**) (Scheme 5). The intermediacy of the intermolecular hydrogen abstraction of N–iron species (**i**) is supported by the fact that the production of **9** was more pronounced in EtOH having a C–H bond α to the oxygen, which likely served as a hydrogen donor (Table 1, entries 1 and 2). It should be mentioned that reduced material **9** may also be produced by Bu_4_NBr alone as observed in our previous study [28]. To elucidate the contribution of this pathway, compound **8** was treated with Bu_4_NX in *t*-BuOH. However, no reduced material was obtained within the reaction times depicted in Table 1 [for instance, 0.5 h stirring for Bu_4_NBr (Table 1, entry 3) and 2.5 h stirring for Bu_4_NCl (Table 1, entry 4)], indicating that the non-iron-mediated process is not significant [42]. Various yields of **9** obtained by loading consistent amounts (1.2–1.5 equiv) of Bu_4_NX salts also indicated the poor contribution of the pathway. Chan and co-workers demonstrated that an iron–imido complex generated from FeCl_2_/PhI=NTs underwent radical hydrogen abstraction from a formyl group, and combined the resultant radicals (hydrogen atom abstraction/radical rebound pathway) to provide amides [43–44]. The involvement of such an iron complex (shown in brackets in Scheme 4) that features radical/metal–nitrenoid properties can be considered in our reactions. A recent study by Betley and co-workers on high-spin iron–imido complexes generated by the reactions of alkyl azides with FeCl_2_ bearing dipyrromethene ligands revealed the radical character of the complex [39–40], harmonizing well with our result, which implies the intermediacy of the nitrogen radical species.

## Conclusion

We have developed a new approach to key compounds **5a**/**5b** for (−)-agelastatin A (**1**) synthesis, which features the iron(II)-mediated radical cyclization of *N*-tosyloxycarbamate, a safe azidoformate surrogate. Although somewhat moderate chemical yields of the compounds were obtained in this study, the elimination of hazardous synthetic processes enables the establishment of more robust strategies to access **1**. Furthermore, the present study has allowed us to obtain mechanistic insights suggesting that N–iron species (**i**) has a metal-radical character. Much work is currently being undertaken to comprehend fully the unique properties of the present reactions.

## Supporting Information

File 1 Experimental procedures, characterization data of new compounds, and ^1^H/^13^C NMR spectra.
