# Supplementary material for: Formal synthesis of (−)-agelastatin A: an iron(II)-mediated cyclization strategy
Source: Beilstein J Org Chem. 2013 May 3;9:860–5. doi: 10.3762/bjoc.9.99 (PMC3678661; doi:10.3762/bjoc.9.99)

Supporting Information

for

Formal synthesis of (−)-agelastatin A: an iron(II)-mediated cyclization strategy

Daisuke Shigeoka, Takuma Kamon and Takehiko Yoshimitsu*

Address: Graduate School of Pharmaceutical Sciences, Osaka University, 1-6 Yamadaoka, Suita, Osaka 565-0871, Japan

Email: Takehiko Yoshimitsu* - yoshimit@phs.osaka-u.ac.jp

* Corresponding author

Experimental procedures, characterization data of new compounds, and $^{1}H/^{13}C$ NMR spectra

Table of Contents

General…………………………………………………………………………….. S3

Preparation of *N*-hydroxycarbamate **7** S3

Preparation of *N*-tosyloxycarbamate **8**... S4

Table 1, entry 4 .... S4

Table 1, entry 1 .... S5

Table 1, entry 2 .... S6

Table 1, entries 3–6 .... S7

Table 1, entry 7 .... S8

Aminobromination of azidoformate **3** with $FeBr_2/Bu_4NBr$ S9

Reaction of enone **10** with TMSCl in EtOH S9

References... S10

Copies of ^{1}H NMR/^{13}C NMR spectra

^{1}H NMR/^{13}C NMR spectra of compound 7 .... S11/S12

^{1}H NMR/^{13}C NMR spectra of compound **8** S13/S14

^{1}H NMR/^{13}C NMR spectra of compound **11** S15/S16

^{1}H NMR/^{13}C NMR spectra of compound **12** S17/S18

^{1}H NMR Spectra of a mixture of compound **5b**, **9**, and **10** (entry 2) S19/S20

^{1}H NMR Spectra of a mixture of compound **5a**, **9**, and **10** (entry 3) S21/S22

^{1}H NMR Spectra of a mixture of compound **5a**, **9**, and **10** (entry 5) S23/S24

^{1}H NMR Spectra of a mixture of compound **5b**, **9**, and **10** (entry 6) S25/S26

^{1}H NMR Spectra of a mixture of compound **5b** and **12** (entry 7) S27/S28

General. Melting points are uncorrected. All reagents were used as received from commercial suppliers unless otherwise noted. ^{1}H NMR spectra (400 MHz) and ^{13}C NMR spectra (100 MHz) were measured in the specified solvents. Chemical shifts are reported in ppm relative to the internal solvent signal [chloroform-*d*: 7.26 ppm (^{1}H NMR), 77. ppm (^{13}C NMR); methanol-*d*: 3.30 ppm (^{1}H NMR), 49.0 ppm (^{13}C NMR); DMSO-d_6: 2.49 ppm (^{1}H NMR), 39.5 ppm (^{13}C NMR)]. FTIR spectra were recorded for samples loaded as neat films on NaCl plates or dispersed in KBr pellets. Mass spectra were obtained according to the specified technique. Analytical thin layer chromatography (TLC) was performed using Kieselgel 60 F_{254}. Compounds were visualized with UV light and stained with anisaldehyde solution or phosphomolybdic acid solution.

Preparation of *N*-tosyloxycarbamate 8 from alcohol (+)-6:

Preparation of *N*-hydroxycarbamate 7: To a stirred solution of alcohol (+)-**6** [1] (300 mg, 1.56 mmol) in THF (14 mL) was added CDI (303 mg, 1.87 mmol). After 4.5 h at room temperature, TLC analysis indicated complete conversion of the starting material into the corresponding imidazolide. To this mixture was added hydroxylamine hydrochloric acid salt (302 mg, 4.68 mmol), and stirring was continued for an additional 23.5 h at room temperature. The reaction mixture was then poured into a separatory funnel where it was partitioned between EtOAc and H_2O. The organic layer was separated, dried over $MgSO_4$, filtered, and concentrated under reduced pressure. The residue was purified by flash silica gel column chromatography (EtOAc → MeOH/EtOAc 1:30 v/v) to afford *N*-hydroxycarbamate **7** (255 mg, 67%) as a colorless solid. $[\alpha]^{22}_{D}$ +105.3 (*c* 0.485, MeOH); IR (KBr) ν 3464, 3246, 1718, 1647 cm^{-1}; ^{1}H NMR (400 MHz, CD_3OD) δ 6.91-6.77 (m, 2H), 6.50 (m, 1H), 6.27-6.16 (m, 2H), 6.09 (m, 1H), 5.79 (m, 1H), 2.51 (ddd, 1H, *J* = 15.2, 8.0, 2.4 Hz), 2.09 (ddd, 1H, *J* = 14.4, 7.6, 2.0 Hz); ^{13}C NMR (100 MHz, CD_3OD) δ 166.6, 160.5, 138.4, 134.9, 125.9, 124.2, 115.6, 109.2, 80.9, 63.1, 41.5; MS *m/z*: 251 (M^+), 174 (100%); HRMS–EI (*m/z*): $[M]^+$ calcd for $C_{11}H_{13}N_3O_4$, 251.0906; found, 251.0922.

Preparation of *N*-tosyloxycarbamate 8: To a solution of *N*-hydroxycarbamate **7** (228 mg, 0.908 mmol) in THF (12.5 mL) at 0 °C were added Et_3N (190 μL, 1.36 mmol) and TsCl (190 mg, 0.998 mmol), and the mixture was warmed to room temperature. After being stirred for 30 min at room temperature, the mixture was poured into a separatory funnel where it was partitioned between EtOAc and H_2O. The phases were separated and the aqueous phase was extracted with EtOAc. The combined organic extracts were dried over $MgSO_4$, filtered, and concentrated under reduced pressure. The residue was purified by flash silica gel column chromatography (EtOAc/*n*-hexane 2:1 v/v) to afford *N*-tosyloxycarbamate **8** (341 mg, 92%) as a colorless solid. $[\alpha]^{20}_{D}$ +71.6 (*c* 0.485, $CHCl_3$); IR (neat) ν 3468, 3374, 2926, 1761, 1645 cm^{-1}; ^1H NMR (400 MHz, $CDCl_3$) δ 9.37 (brs, 1H), 7.89 (d, 2H, *J* = 8.2 Hz), 7.37 (d, 2H, *J* = 8.7 Hz), 6.75-6.61 (m, 2H), 6.42 (m, 1H), 6.19-6.06 (m, 2H), 6.03 (m, 1H), 5.92-5.52 (m, 3H), 2.46 (s, 3H), 2.36 (ddd, 1H, *J* = 15.1, 7.8, 2.7 Hz), 2.04 (ddd, 1H, *J* = 15.1, 7.3, 4.1 Hz), ^{13}C NMR (100 MHz, $CDCl_3$) δ 163.6, 155.4, 146.1, 137.4, 133.4, 130.3, 129.7, 129.6, 124.0, 123.3, 114.0, 108.3, 81.2, 61.8, 39.8, 21.8; MS *m/z*: 406 (MH^+), 154 (100%); HRMS–FAB $[M + H]^+$ calcd for $C_{18}H_{20}N_3O_6S$, 406.1073; found, 406.1073.

Protocols for aminohalogenation of *N*-tosyloxycarbamate 8 with FeX_2/Bu_4NX:

Table 1, entry 4

To a solution of *N*-tosyloxycarbamate **8** (20 mg, 0.049 mmol) in *t*-BuOH (1 mL) were added Bu_4NCl (16.4 mg, 0.059 mmol) and $FeCl_2$ (3.2 mg, 0.025 mmol). The mixture was warmed to 28 °C in a water bath and sonicated for ca. 1 min to dissolve all the reagents. After being stirred for 2.5 h at the same temperature, the mixture was poured into a separatory funnel where it was partitioned between EtOAc and H_2O. The phases were separated, and the aqueous phase was extracted with EtOAc. The combined organic extracts were dried over $MgSO_4$, filtered, and concentrated under reduced pressure. The residue was purified by flash silica gel column chromatography (EtOAc/*n*-hexane 2:1→3:1→EtOAc v/v) to afford chloride **5b** (6.3 mg, 48%) carbamate **9** (1.2 mg, 9%), and enone **10** (0.9 mg, 9%), all as colorless solids. **Chloride 5b**: IR (KBr) ν 3339, 2928, 1746, 1653 cm^{-1}; ^1H NMR (400 MHz, CD_3OD) δ 7.15 (dd, 1H, *J* = 2.8, 1.8 Hz), 6.81 (dd, 1H, *J* = 3.7, 1.8 Hz), 6.19 (dd, 1H, *J* = 3.7, 2.8 Hz), 5.92 (ddd, 1H, *J* = 11.5, 11.4,

6.9 Hz), 5.15 (t, 1H, J = 6.9 Hz), 4.55 (dd, 1H, J = 11.5, 6.0 Hz), 4.46 (m, 1H), 2.54 (dd, 1H, J = 14.7, 6.9 Hz), 2.31 (ddd, 1H, J = 14.7, 11.4, 6.0 Hz); ^{1}H NMR (400 MHz, DMSO-d_6) δ 8.14 (d, 1H, J = 0.9 Hz), 7.55 (brs, 1H), 7.23 (dd, 1H, J = 2.8, 1.8 Hz), 6.93 (brs, 1H), 6.79 (dd, 1H, J = 3.6, 1.8 Hz), 6.12 (dd, 1H, J = 3.6, 2.8 Hz), 5.95 (ddd, 1H, J = 11.9, 11.4, 7.3 Hz), 5.05 (dd, 1H, J = 6.9, 6.8 Hz), 4.67 (dd, 1H, J = 11.4, 5.5 Hz), 4.37 (m, 1H), 2.31 (dd, 1H, J = 13.7, 7.3 Hz), 2.19 (ddd, 1H, J = 13.7, 11.9, 6.0 Hz), ^{13}C NMR (125 MHz, DMSO-d_6) δ 163.1, 158.5, 126.3, 122.7, 113.6, 108.3, 75.9, 63.9, 58.5, 56.9, 37.7. **Carbamate 9**: IR (KBr) ν 3389, 2951, 1690, 1663 cm^{-1}; ^{1}H NMR (400 MHz, CD_3OD) δ 6.86-6.77 (m, 2H), 6.49 (m, 1H), 6.21-6.13 (m, 2H), 6.08 (dd, 1H, J = 3.7, 2.7 Hz), 5.69 (m, 1H), 2.49 (ddd, 1H, J = 15.1, 7.8, 2.7 Hz), 2.06 (ddd, 1H, J = 15.1, 7.3, 4.6 Hz); ^{13}C NMR (100 MHz, CD_3OD) δ 166.6, 159.7, 137.9, 135.3, 125.9, 124.2, 115.6, 109.1, 80.1, 63.1, 41.5. **Enone 10**: IR (neat) ν 3349, 2926, 1715, 1645 cm^{-1}; ^{1}H NMR (400 MHz, CD_3OD) δ 7.76 (dd, 1H, J = 5.5, 2.7 Hz), 6.90-6.80 (m, 2H), 6.66 (m, 1H), 6.39 (dd, 1H, J = 5.5, 2.3 Hz), 6.15 (dd, 1H, J = 4.1, 2.8 Hz), 3.02 (dd, 1H, J = 18.8, 6.4 Hz), 2.27 (dd, 1H, J = 18.8, 2.3 Hz); ^{1}H NMR (300 MHz, $CDCl_3$) δ 7.65 (dd, 1H, J = 5.9, 2.4 Hz), 6.86-6.72 (m, 3H), 6.42 (dd, 1H, J = 5.9, 1.7 Hz), 6.19 (dd, 1H, J = 3.7, 2.8 Hz), 5.60 (brs, 2H), 3.12 (dd, 1H, J = 19.3, 6.5 Hz), 2.31 (dd, 1H, J = 19.3, 2.4 Hz); ^{13}C NMR (100 MHz, CD_3OD) δ 208.9, 166.3, 163.1, 136.7, 126.0, 124.7, 115.7, 109.9, 58.1, 45.0. All the spectroscopic and analytical data of chloride **5b**, carbamate **9**, and enone **10** were identical with those reported in the previous study [2]. The chemical shifts in the ^{13}C NMR spectrum of carbamate **9** in the literature [2] were erroneous due to the incorrect assignment of reference solvent signal (CD_3OD for 49.0 ppm). The corrected data are given above.

Table 1, entry 1

To a solution of *N*-tosyloxycarbamate **8** (20 mg, 0.049 mmol) in EtOH (1 mL) were added Bu_4NBr (23.8 mg, 0.074 mmol) and $FeBr_2$ (5.4 mg, 0.025 mmol). The mixture was warmed to 28 °C in a water bath and sonicated for ca. 1 min to dissolve all the reagents. After being stirred for 1.75 h at the same temperature, the mixture was poured into a separatory funnel where it was partitioned between EtOAc and H_2O. The phases were separated, and the aqueous phase was extracted with EtOAc. The combined organic

extracts were dried over $MgSO_4$, filtered, and concentrated under reduced pressure. The residue was purified by flash silica gel column chromatography (EtOAc/*n*-hexane 2:1→EtOAc v/v) to afford bromide **5a** (2.0 mg, 13%), enone **10** (2.8 mg, 30%), and carbamate **9** (4.5 mg, 39%). **Bromide 5b**: IR (KBr) ν 3376, 2955, 1751, 1647 cm^{-1}; ^1H NMR (400 MHz, CD_3OD) δ 7.13 (dd, 1H, *J* = 2.8, 1.4 Hz), 6.81 (dd, 1H, *J* = 3.7, 1.4 Hz), 6.19 (dd, 1H, *J* = 3.7, 2.8 Hz), 5.97 (ddd, 1H, *J* = 11.5, 11.0, 7.3 Hz), 5.17 (t, 1H, *J* = 6.9 Hz), 4.57 (dd, 1H, *J* = 11.0, 6.0 Hz), 4.47 (m, 1H), 2.55 (dd, 1H, *J* = 14.2, 7.3 Hz), 2.29 (ddd, 1H, *J* = 14.2, 11.5, 6.0 Hz); ^1H NMR (400 MHz, DMSO-d_6) δ 8.11 (d, 1H, *J* = 0.9 Hz), 7.55 (brs, 1H), 7.21 (dd, 1H, *J* = 2.7, 1.4 Hz), 6.92 (brs, 1H), 6.78 (dd, 1H, *J* = 3.7, 1.4 Hz), 6.11 (dd, 1H, *J* = 3.7, 2.7 Hz), 6.00 (ddd, 1H, *J* = 12.4, 11.4, 7.3 Hz), 5.06 (dd, 1H, *J* = 6.9, 6.4 Hz), 4.69 (dd, 1H, *J* = 11.4, 5.5 Hz), 4.37 (m, 1H), 2.31 (dd, 1H, *J* = 13.7, 7.3 Hz), 2.15 (ddd, 1H, *J* = 13.7, 12.4, 6.4 Hz), ^{13}C NMR (125 MHz, DMSO-d_6) δ 163.1, 158.4, 126.3, 122..6, 113.5, 108.3, 76.3, 58.8, 57.1, 56.0, 38.0. The spectroscopic and analytical data of bromide **5a** were exactly matched with those reported in the previous study [2].

Table 1, entry 2

To a solution of *N*-tosyloxycarbamate **8** (20 mg, 0.0493 mmol in EtOH (1 mL) were added Bu_4NCl (16.4 mg, 0.0592 mmol) and $FeCl_2$ (3.2 mg, 0.0247 mmol). The mixture was warmed to 28 °C in a water bath and sonicated for ca. 1 min to dissolve all the reagents. After being stirred for 0.75 h at the same temperature, the mixture was poured into a separatory funnel where it was partitioned between EtOAc and H_2O. The phases were separated, and the aqueous phase was extracted with EtOAc. The combined organic extracts were dried over $MgSO_4$, filtered, and concentrated under reduced pressure. The residue was purified by flash silica gel column chromatography (EtOAc/*n*-hexane 2:1→EtOAc v/v) to afford chloride **5b** (5.2 mg, 39%), enone **10** (1.8 mg, 19%), and carbamate **9** (2.4 mg, 20%). The yields of the materials were determined by ^1H NMR analysis.

Table 1, entry 3

To a solution of *N*-tosyloxycarbamate **8** (20 mg, 0.049 mmol in *t*-BuOH (1 mL) were added Bu_4NBr (19.1 mg, 0.059 mmol) and $FeBr_2$ (5.4 mg, 0.025 mmol). The mixture was warmed to 28 °C in a water bath and sonicated for ca. 1 min to dissolve all the reagents. After being stirred for 0.5 h at the same temperature, the mixture was poured into a separatory funnel where it was partitioned between EtOAc and H_2O. The phases were separated, and the aqueous phase was extracted with EtOAc. The combined organic extracts were dried over $MgSO_4$, filtered, and concentrated under reduced pressure. The residue was purified by flash silica gel column chromatography (EtOAc/*n*-hexane 2:1→EtOAc v/v) to afford bromide **5a** (5.8 mg, 38%), carbamate **9** (2.5 mg, 22%), and enone **10** (1.8 mg, 19%). The yields of the materials were determined by ^{1}H NMR analysis.

Table 1, entry 5

To a solution of *N*-tosyloxycarbamate **8** (20 mg, 0.049 mmol) in *t*-BuOH (1 mL) were added Bu_4NBr (19.1 mg, 0.059 mmol) and $FeBr_2$ (2.2 mg, 0.01 mmol). The mixture was warmed to 28 °C in a water bath and sonicated for ca. 1 min to dissolve all the reagents. After being stirred for 3.3 h at the same temperature, the mixture was poured into a separatory funnel where it was partitioned between EtOAc and H_2O. The phases were separated, and the aqueous phase was extracted with EtOAc. The combined organic extracts were dried over $MgSO_4$, filtered, and concentrated under reduced pressure. The residue was purified by flash silica gel column chromatography (EtOAc/*n*-hexane 2:1 v/v → EtOAc) to afford bromide **5a** (3.8 mg, 25%), carbamate **9** (1.9 mg, 16%), and enone **10** (0.5 mg, 5%), and unreacted *N*-tosyloxycarbamate **8** (4.4 mg, 22%). The yields of the materials were determined by ^{1}H NMR analysis.

Table 1, entry 6

To a solution of *N*-tosyloxycarbamate **8** (20 mg, 0.049 mmol) in *t*-BuOH (1 mL) were added Bu_4NCl (16.4 mg, 0.059 mmol) and $FeCl_2$ (1.3 mg, 0.01 mmol). The mixture was warmed to 28 °C in a water bath and sonicated for ca. 1 min to dissolve all the reagents. After being stirred for 3.3 h at the same temperature, the mixture was poured into a

separatory funnel where it was partitioned between EtOAc and H_2O. The phases were separated, and the aqueous phase was extracted with EtOAc. The combined organic extracts were dried over $MgSO_4$, filtered, and concentrated under reduced pressure. The residue was purified by flash silica gel column chromatography (EtOAc/*n*-hexane 2:1 v/v → EtOAc) to afford chloride **5b** (4.1 mg, 31%), carbamate **9** (1.1 mg, 9%), and enone **10** (1.3 mg, 14%), and unreacted *N*-tosyloxycarbamate **8** (6.0 mg, 30%). The yields of the materials were determined by ^{1}H NMR analysis.

Table 1, entry 7

To a solution of *N*-tosyloxycarbamate **8** (20 mg, 0.049 mmol) in EtOH (1 mL) were added TMSCl (9.4 µL, 0.073 mmol) and $FeCl_2$ (3.2 mg, 0.025 mmol). The mixture was warmed to 28 °C in a water bath and sonicated for ca. 1 min to dissolve all the reagents. After being stirred for 16 h at the same temperature, the mixture was poured into a separatory funnel where it was partitioned between EtOAc and H_2O. The phases were separated, and the aqueous phase was extracted with EtOAc. The combined organic extracts were dried over $MgSO_4$, filtered, and concentrated under reduced pressure. The residue was purified by flash silica gel column chromatography (EtOAc/*n*-hexane 4:1→ EtOAc) to afford chloride **5b** (3.9 mg, 29%), carbamate **9** (1.4 mg, 12%), diethyl ketal **12** (1.8 mg, 14%), and ketone **11** (1.5 mg, 16%). The yields of the materials were determined by ^{1}H NMR analysis. **Ketone 11:** colorless solid; $[\alpha]^{21}_{D}$ −10.1 (*c* 0.14, MeOH); IR (neat) ν 3223, 2926, 1748, 1645 cm^{-1}; ^{1}H NMR (400 MHz, CD_3OD) δ 7.05 (dd, 1H, *J* = 2.7, 1.4 Hz), 6.91 (dd, 1H, *J* = 3.7, 1.4 Hz), 6.27 (dd, 1H, *J* = 3.7, 2.7 Hz), 4.95 (ddd, 1H, *J* = 12.4, 7.3, 5.0 Hz), 4.53 (m, 1H), 2.84 (dd, 1H, *J* = 18.3, 7.3 Hz), 2.71 (dd, 1H, *J* = 18.3, 6.0 Hz), 2.57 (ddd, 1H, *J* = 18.7, 7.3, 1.8 Hz), 2.44 (ddd, 1H, *J* = 18.7, 3.7, 1.4 Hz); ^{13}C NMR (100 MHz, CD_3OD) δ 213.0, 162.4, 125.4, 122.9, 115.6, 111.4, 54.9, 53.4, 45.4, 43.7; MS *m/z*: 190 (M^+), 190 (100%); HRMS–EI (*m*/*z*): $[M]^+$ calcd for $C_{10}H_{10}N_2O_2$, 190.0742; found, 190.0741. **Diethyl ketal 12:** colorless solid; $[\alpha]^{22}_{D}$ +1.8 (*c* 0.18, $CHCl_3$); IR (neat) ν 3206, 2974, 1651 cm^{-1}; ^{1}H NMR (400 MHz, $CDCl_3$) δ 6.97 (dd, 1H, *J* = 3.7, 1.4 Hz), 6.44 (dd, 1H, *J* = 2.9, 1.4 Hz), 6.27 (dd, 1H, *J* = 3.7, 1.4 Hz), 5.92 (brs, 1H), 4.56 (m, 1H), 4.26 (m, 1H), 3.55-3.33 (m, 4H), 2.47 (dd, 1H, *J* = 13.3, 6.9 Hz), 2.42-2.29 (m, 2H), 2.02 (dd, 1H, *J* = 13.7, 5.0 Hz), 1.20 (t, 3H, *J* = 6.9 Hz), 1.12

(t, 3H, J = 6.9 Hz); ^{13}C NMR (100 MHz, $CDCl_3$) δ 159.5, 122.8, 122.3, 114.0, 110.2, 107.5, 57.8, 56.4, 55.0, 53.4, 42.3, 41.6, 15.3, 15.1; MS *m/z*: 264 (M^+), 135 (100%); HRMS–EI (*m/z*): $[M]^+$ calcd for $C_{14}H_{20}N_2O_3$, 264.1474; found, 264.1475.

Aminobromination of azidoformate 3 with $FeBr_2$/Bu_4NBr: To a stirred solution of azidoformate **3** (2.0 g, 7.66 mmol) in EtOH (100 mL) were added Bu_4NBr (3.7 g, 11.4 mmol) and $FeBr_2$ (843 mg, 3.81 mmol). The mixture was warmed to 28 °C in a water bath and sonicated for ca. 1 min to dissolve all the reagents. After being stirred for 10 h at the same temperature, the mixture was filtered through a pad of celite/florisil, and the filtrate was concentrated under reduced pressure. The residue was diluted with EtOAc and poured into a separatory funnel where it was partitioned between EtOAc and H_2O. The organic extracts were combined, dried over $MgSO_4$, filtered, and concentrated. The residue was rinsed with MeOH and filtered to give bromide **5a** (1.61 g, 67%) as a colorless solid. The filtrate was concentrated under reduced pressure to provide a residue, which was further purified by flash silica gel column chromatography (EtOAc/*n*-hexane 4:1 v/v → EtOAc) to afford carbamate **9** (281 mg, 16%), enone **10** (51 mg, 3%), and additional bromide **5a** (67 mg, 3%), all as colorless solids.

Reaction of enone 10 with TMSCl in EtOH: To a solution of enone **10** (15 mg, 0.08 mmol) in EtOH (1 mL) was added TMSCl (12 µL, 0.09 mmol). After being stirred for 3 h at room temperature, the mixture was poured into a separatory funnel where it was partitioned between EtOAc and H_2O. The phases were separated and the aqueous phase was extracted with EtOAc. The combined organic extracts were dried over $MgSO_4$, filtered, and concentrated under reduced pressure. The residue was purified by flash silica gel column chromatography (EtOAc/*n*-hexane 4:1→6:1→EtOAc v/v) to give ketone **11** (1.5 mg, 10%) and diethyl ketal **12** (4.0 mg, 19%), both as colorless solids.

References

1) Yoshimitsu, T.; Ino, T.; Tanaka, T. *Org. Lett.* **2008**, *10*, 5457.

2) Yoshimitsu, T.; Ino, T.; Futamura, N.; Kamon, T.; Tanaka, T. *Org. Lett.* **2009**, *11*, 3402.

400MHz, CD_3OD

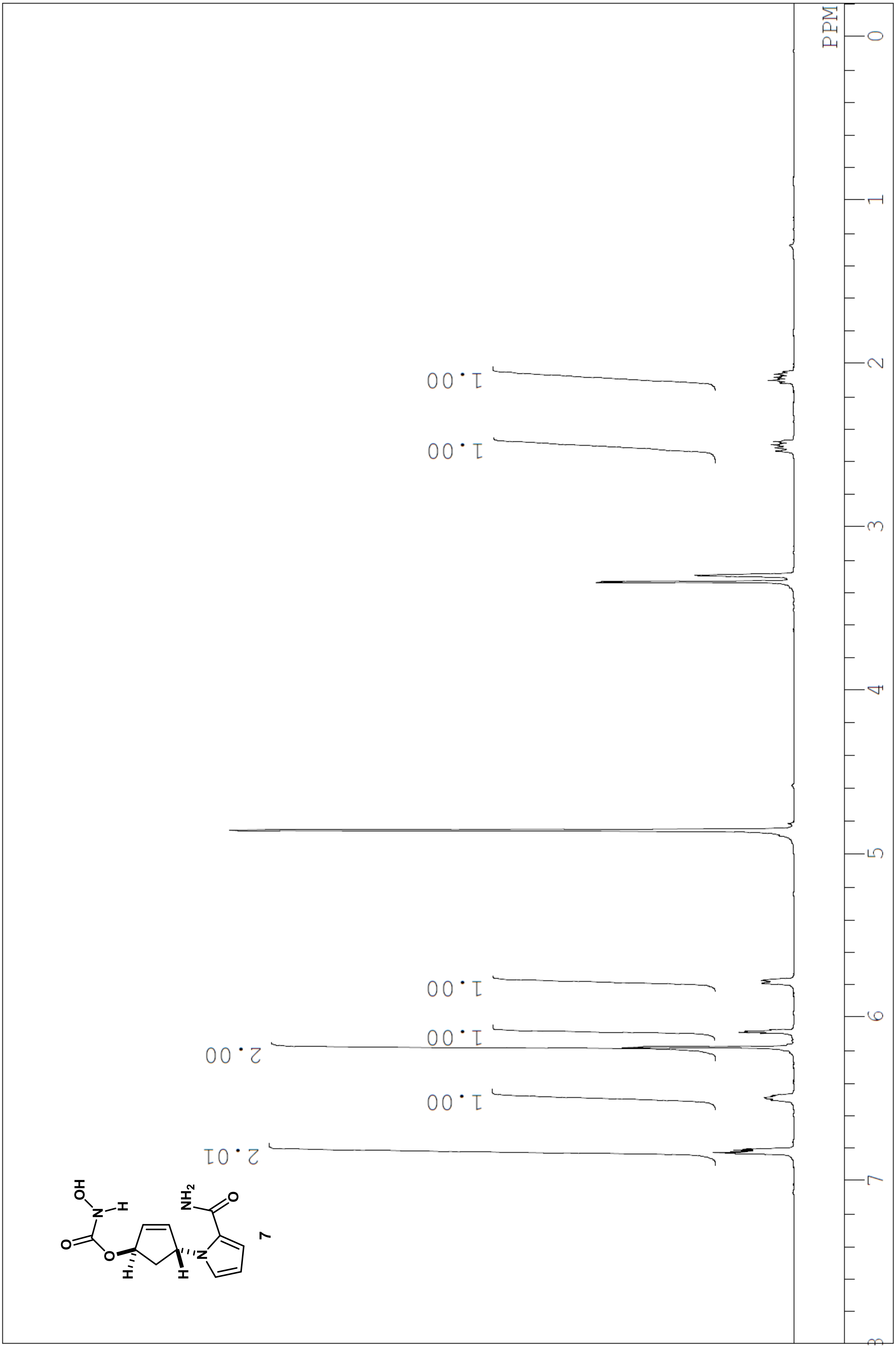

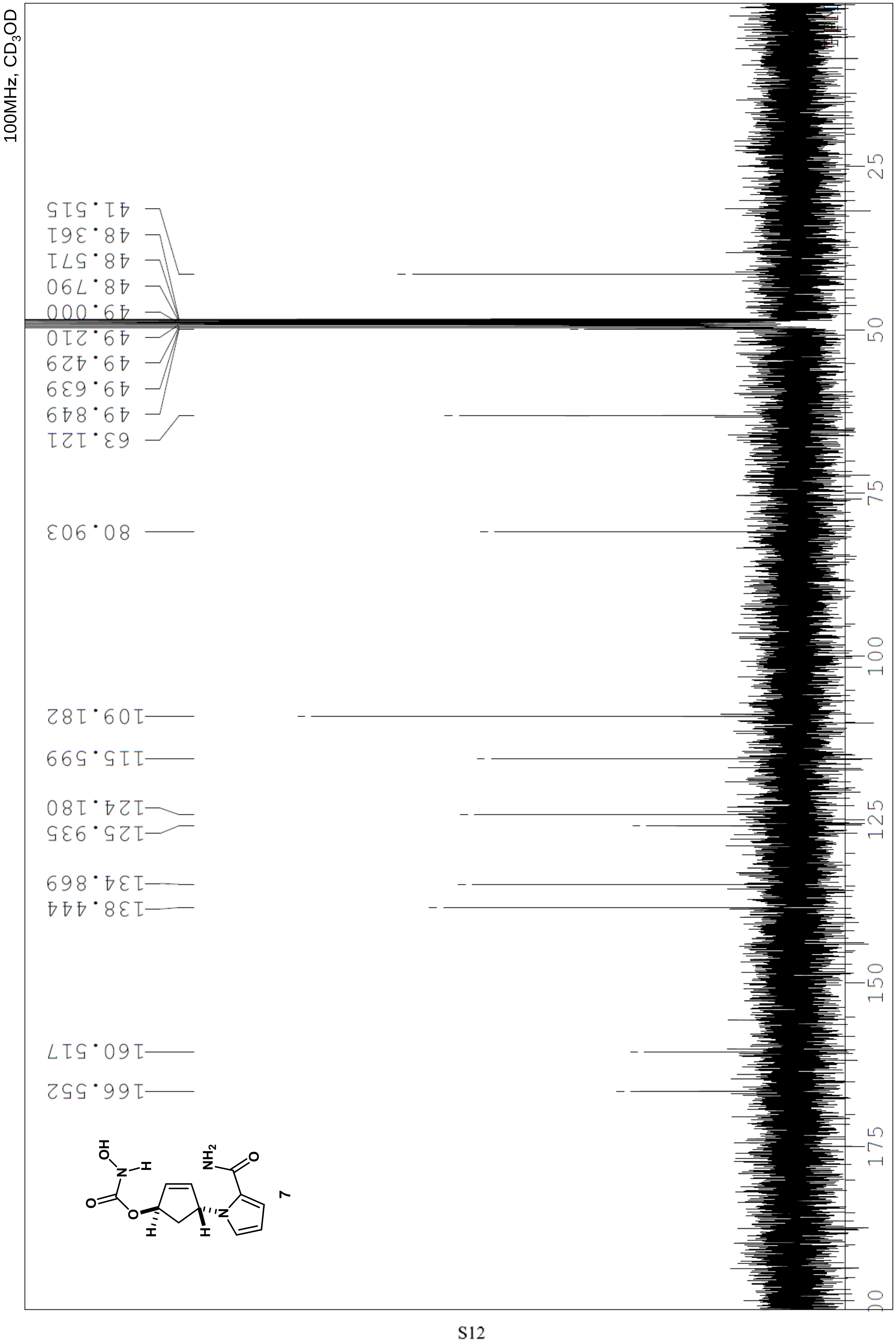
100MHz, CD3OD
41.515
48.361
48.571
48.790
49.000
49.210
49.429
49.639
49.849
63.121
80.903
109.182
115.599
124.180
125.935
134.869
138.444
160.517
166.552
25
50
75
100
125
150
175
OH
N
H
O
O
NH2
O
N
H
H
7

400MHz, $CDCl_3$

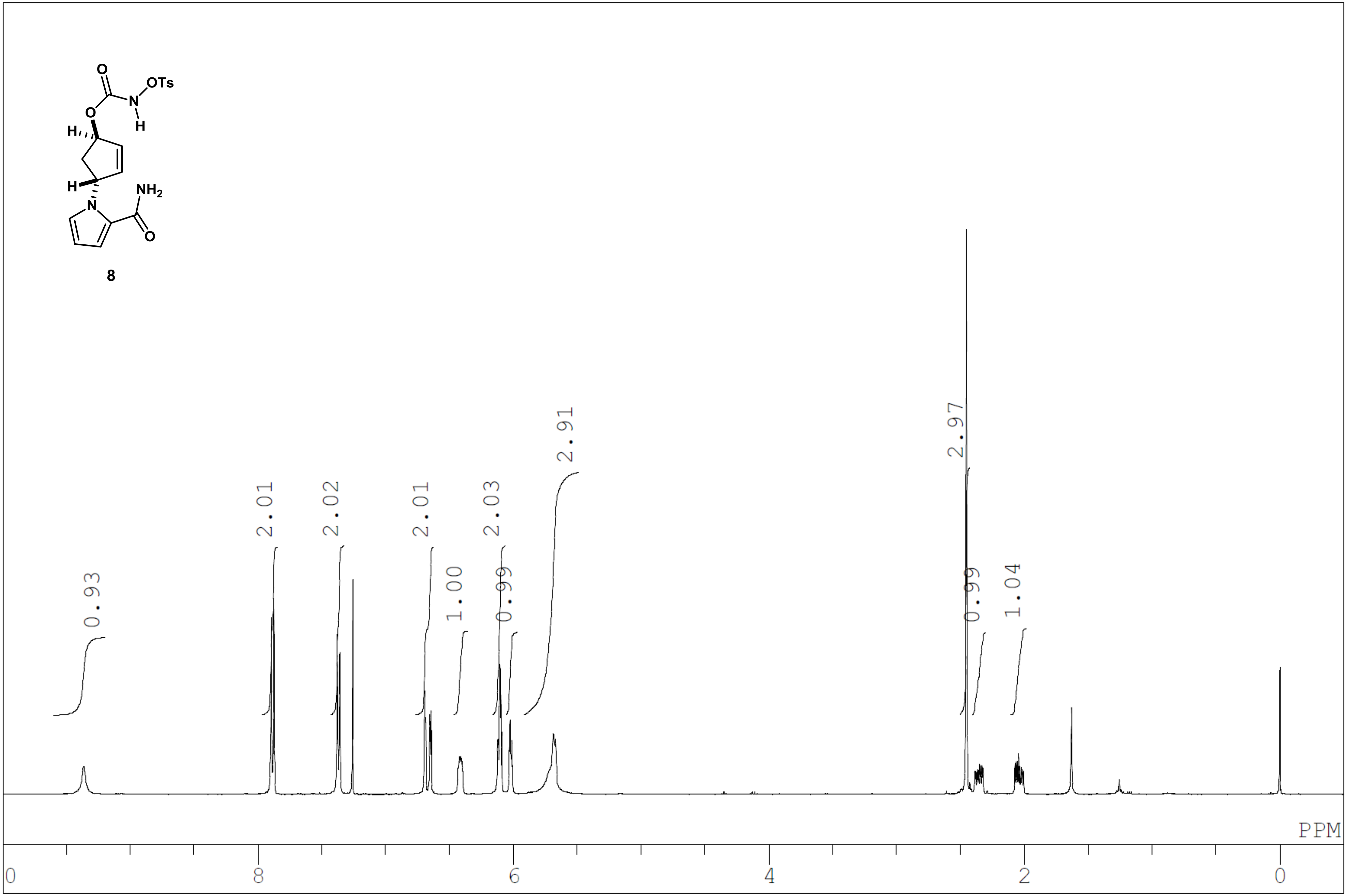

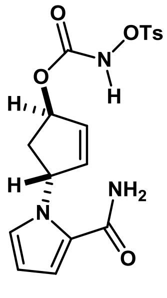

8

100MHz, $CDCl_3$

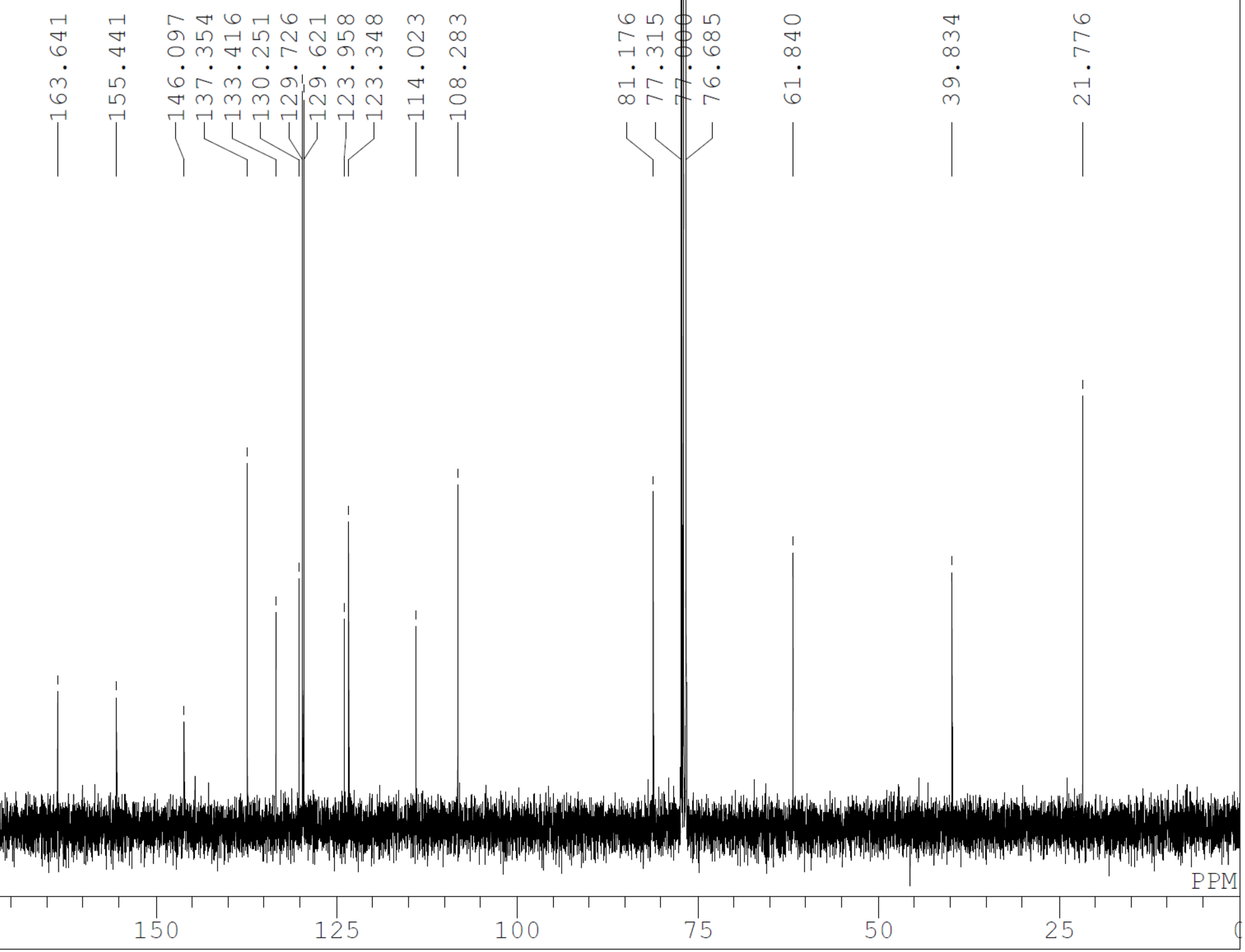

400MHz, CD_3OD

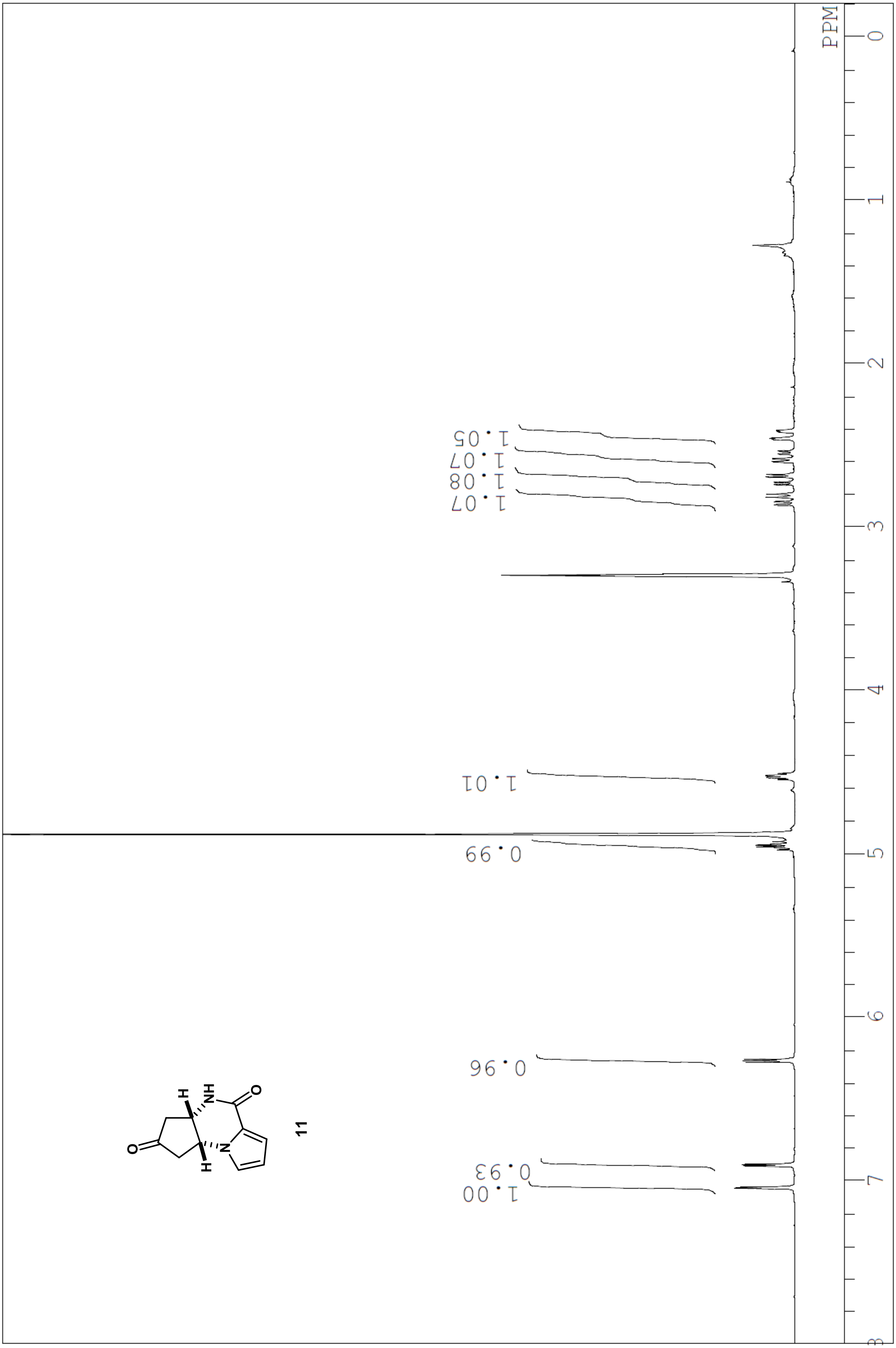

100MHz, CD_3OD

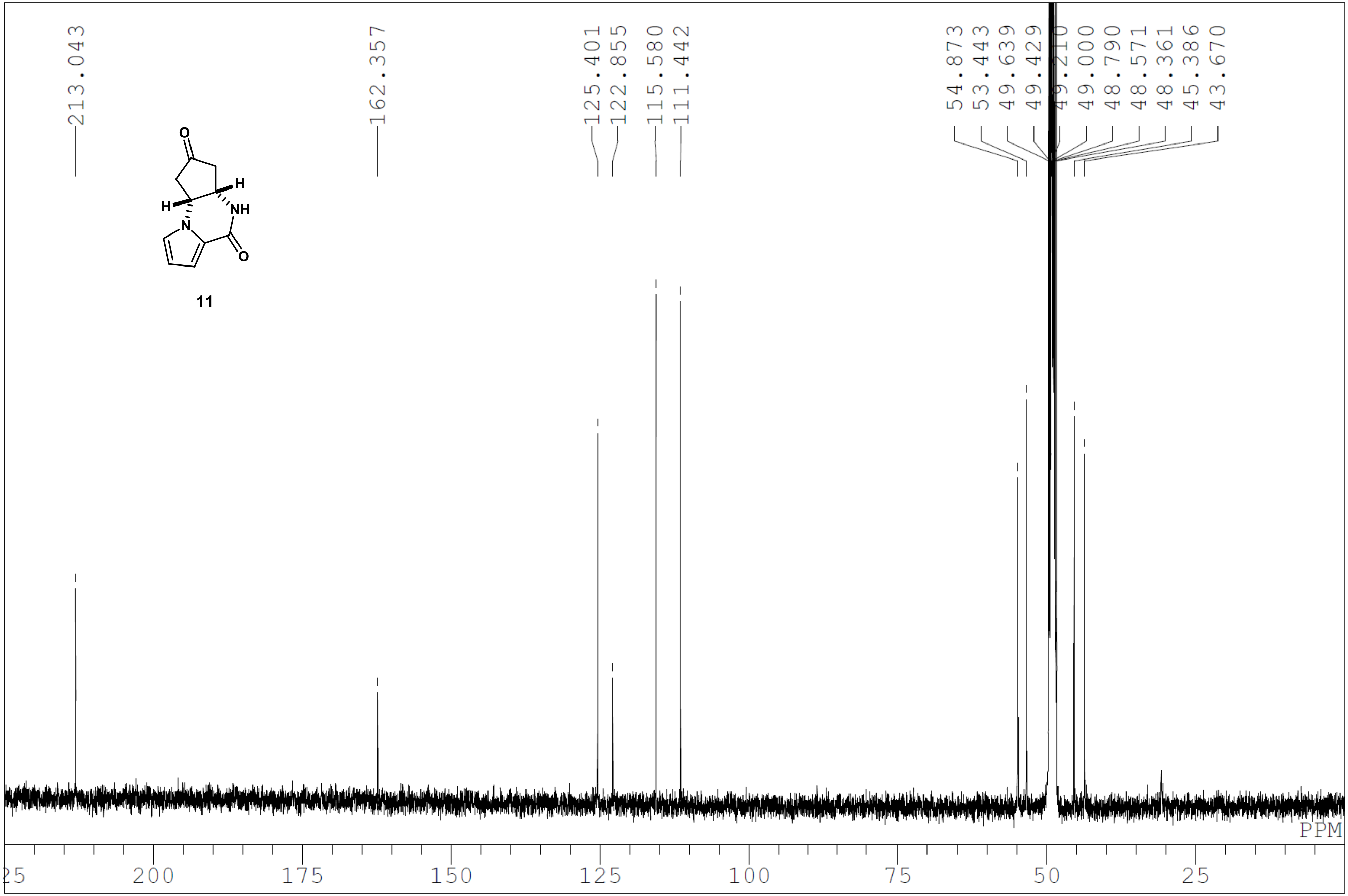

11

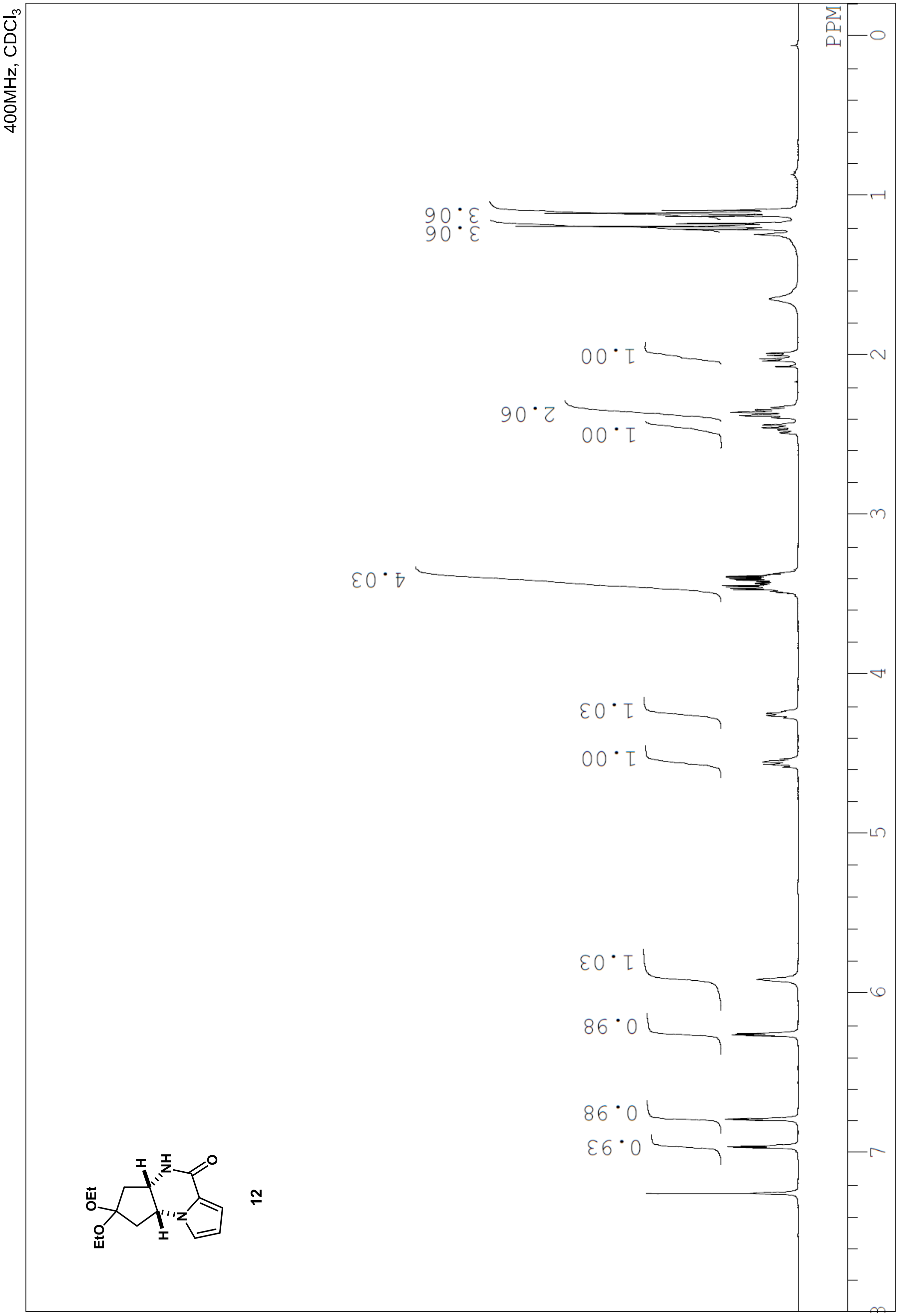
400MHz, CDCl3
PPM
0
1
2
3
4
5
6
7
3.06
3.06
1.00
2.06
1.00
4.03
1.03
1.00
1.03
0.98
0.98
0.93
OEt
EtO
H
NH
O
N
H
12

100MHz, $CDCl_3$

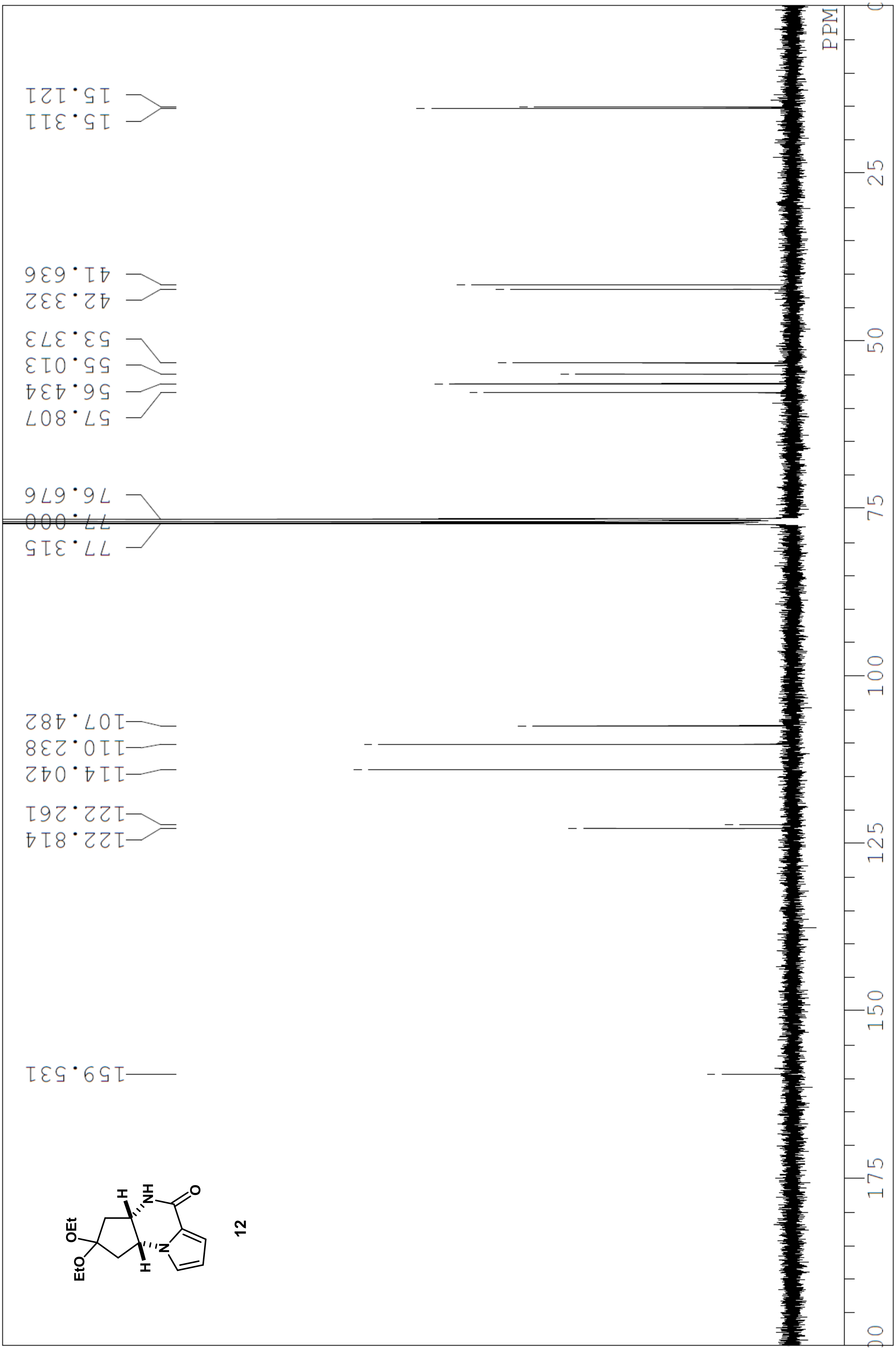

Table 1, entry 2

400MHz, CD_3OD

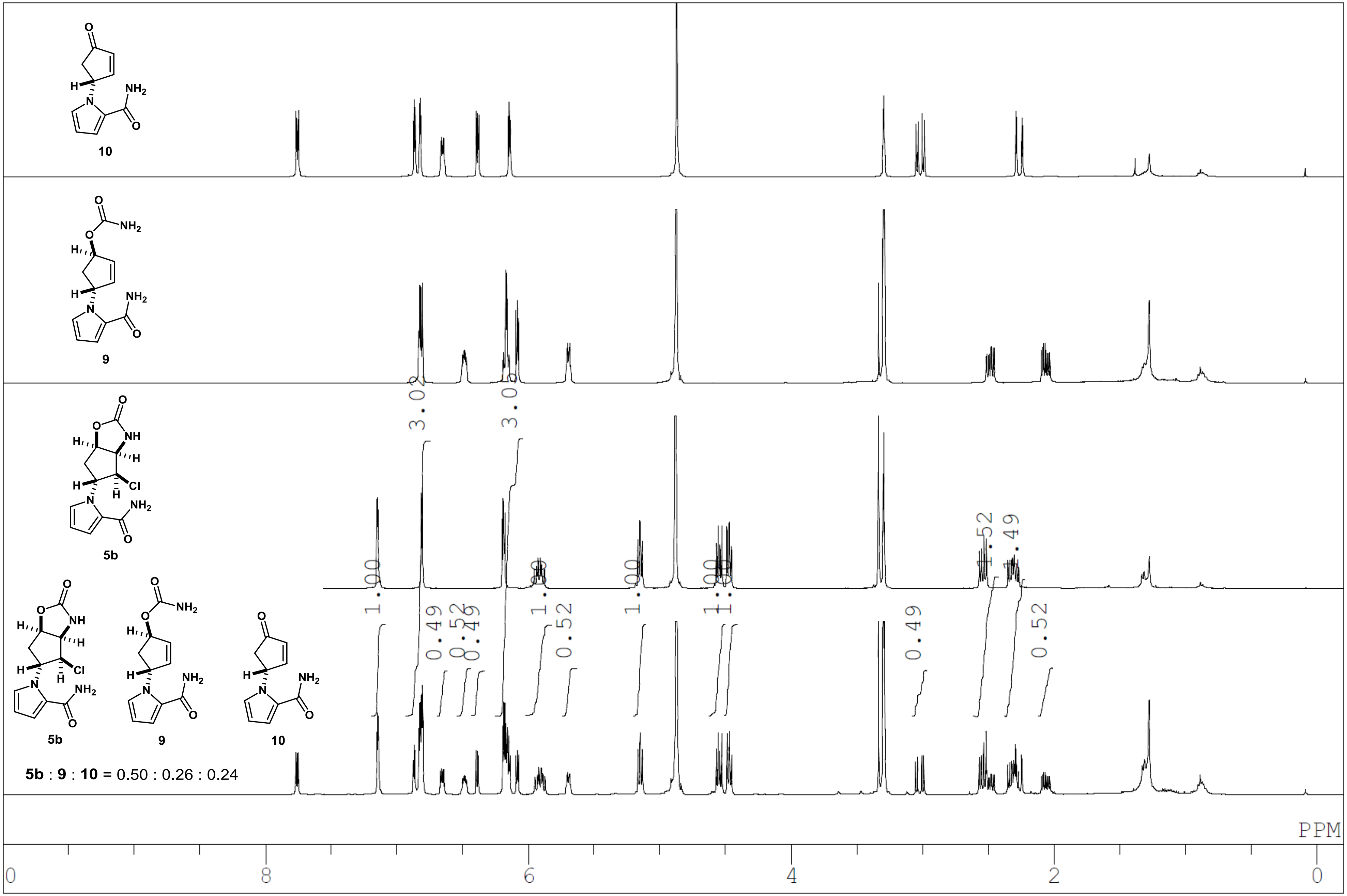

Table 1, entry 2

400MHz, CD_3OD

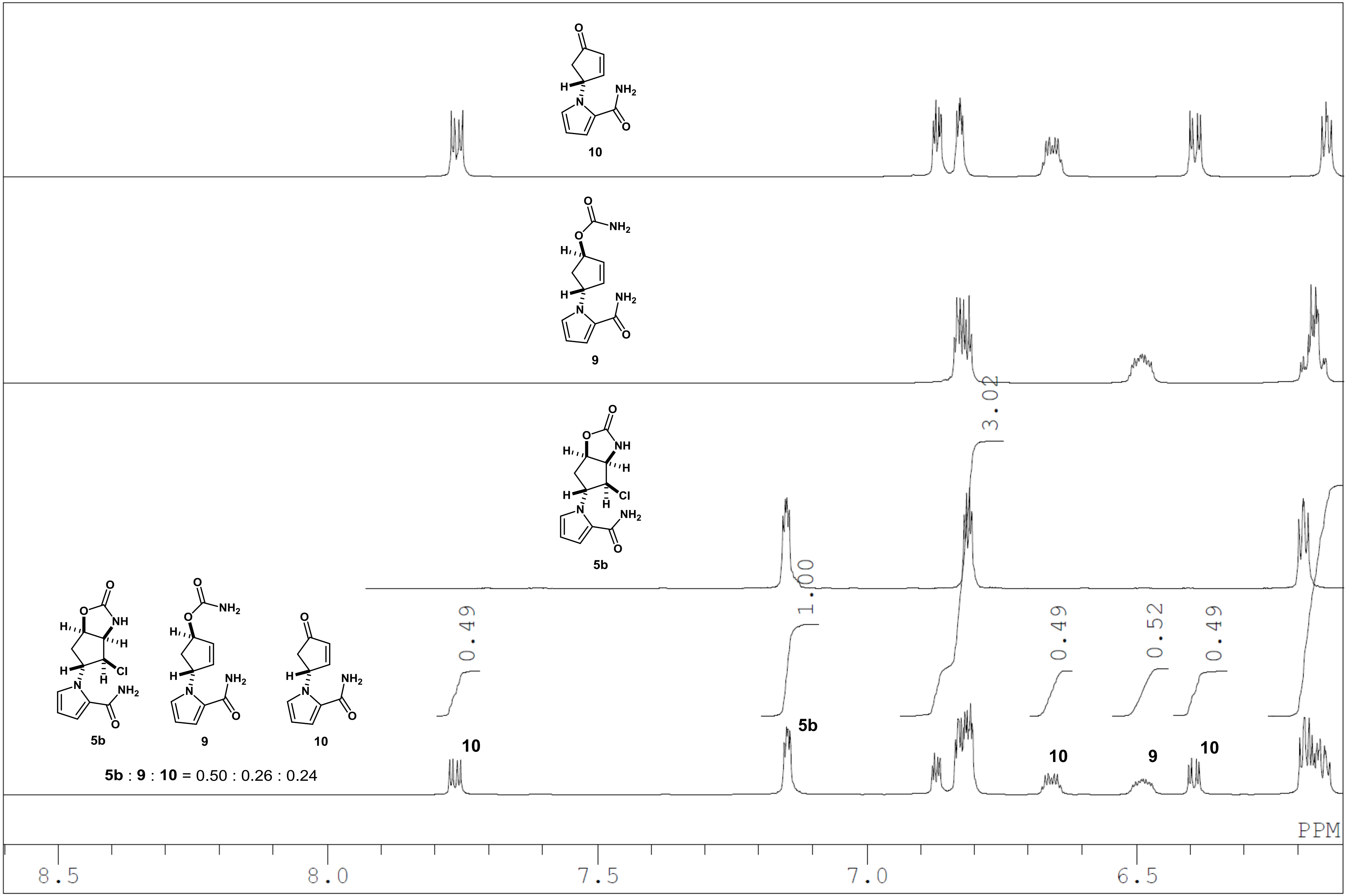

Table 1, entry 3

400MHz, CD_3OD

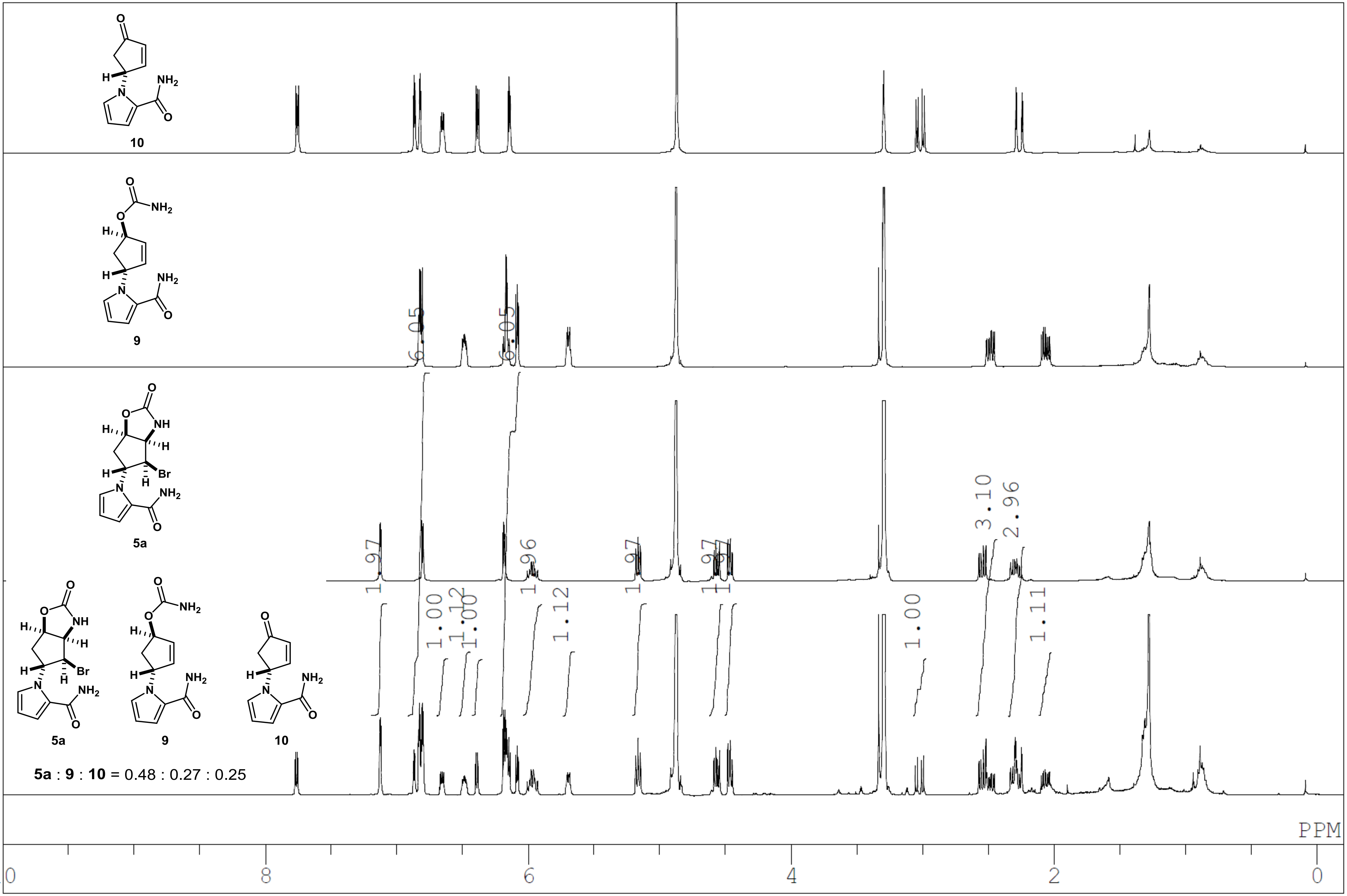

5a : **9** : **10** = 0.48 : 0.27 : 0.25

Table 1, entry 3

400MHz, CD_3OD

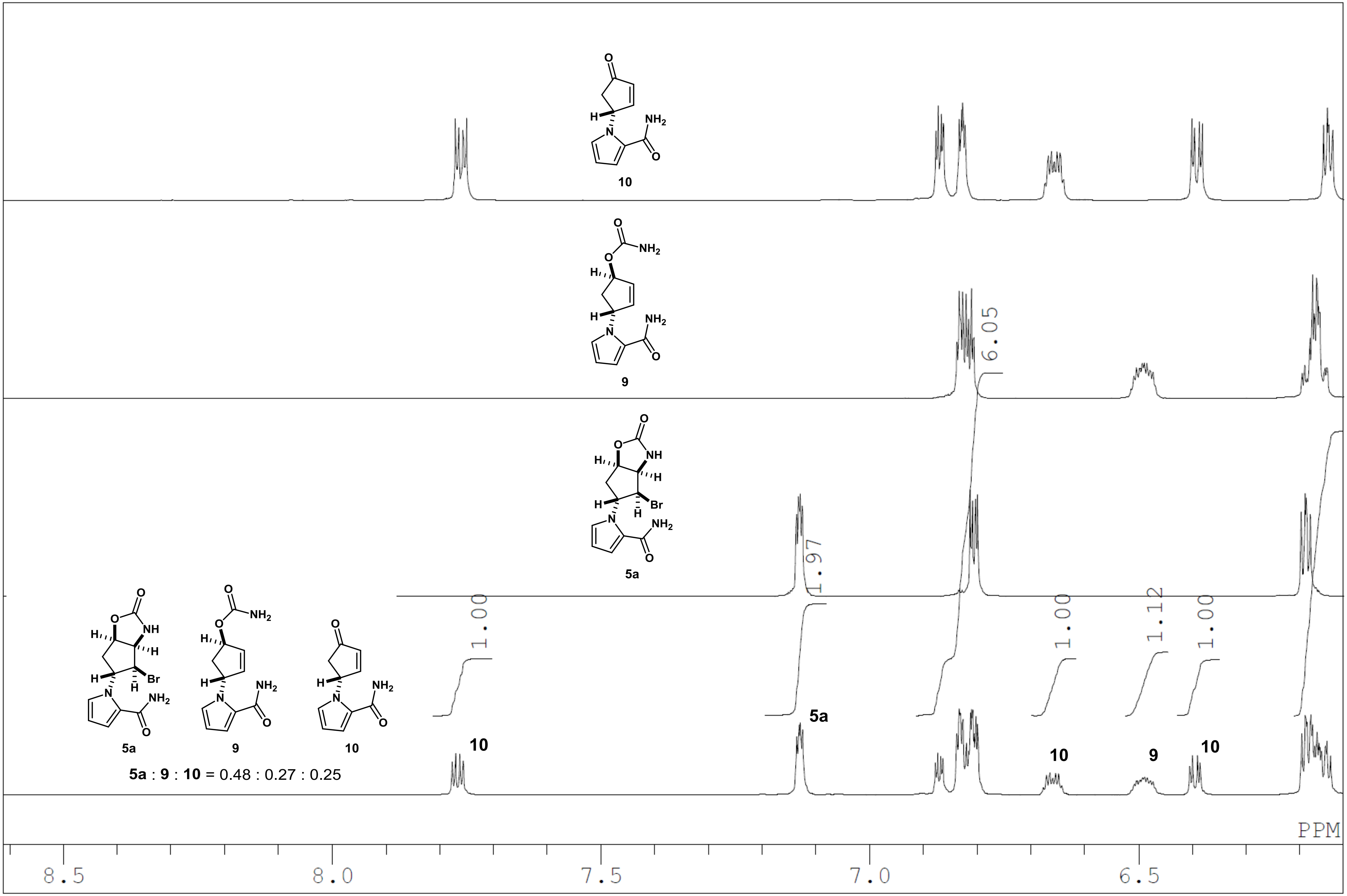

Table 1, entry 5

400MHz, CD_3OD

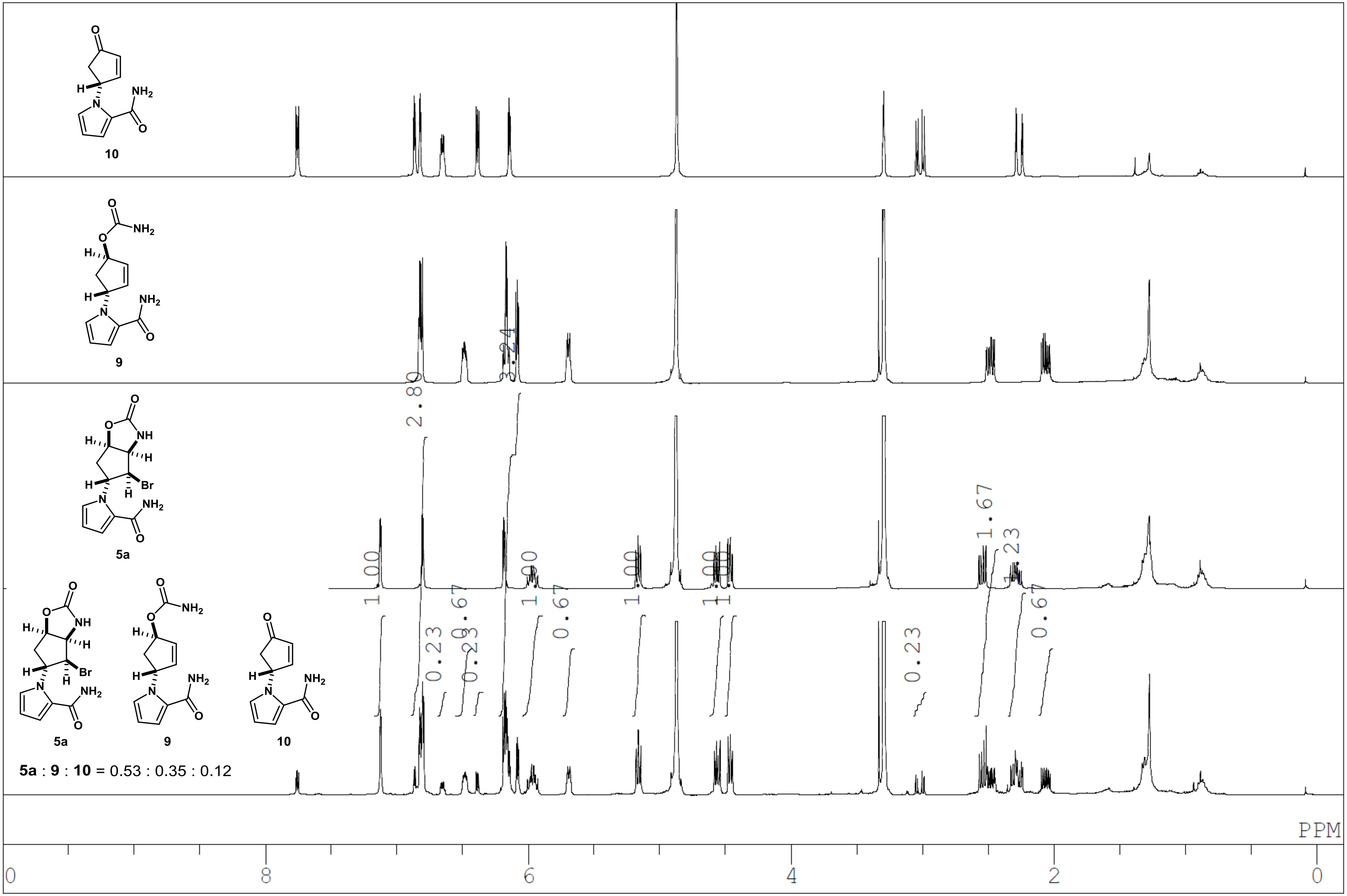

Table 1, entry 5

400MHz, CD_3OD

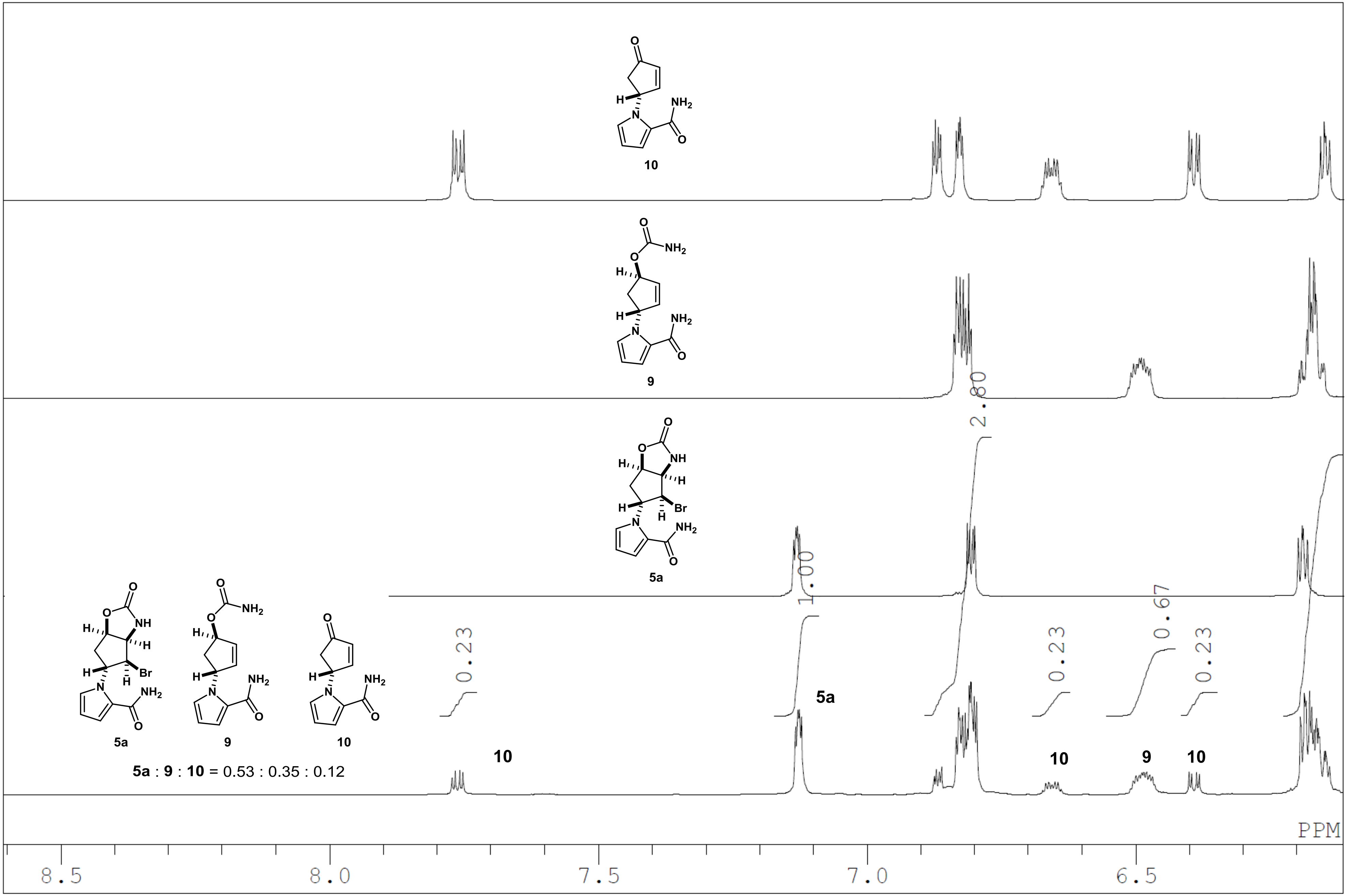

Table 1, entry 6

400MHz, CD_3OD

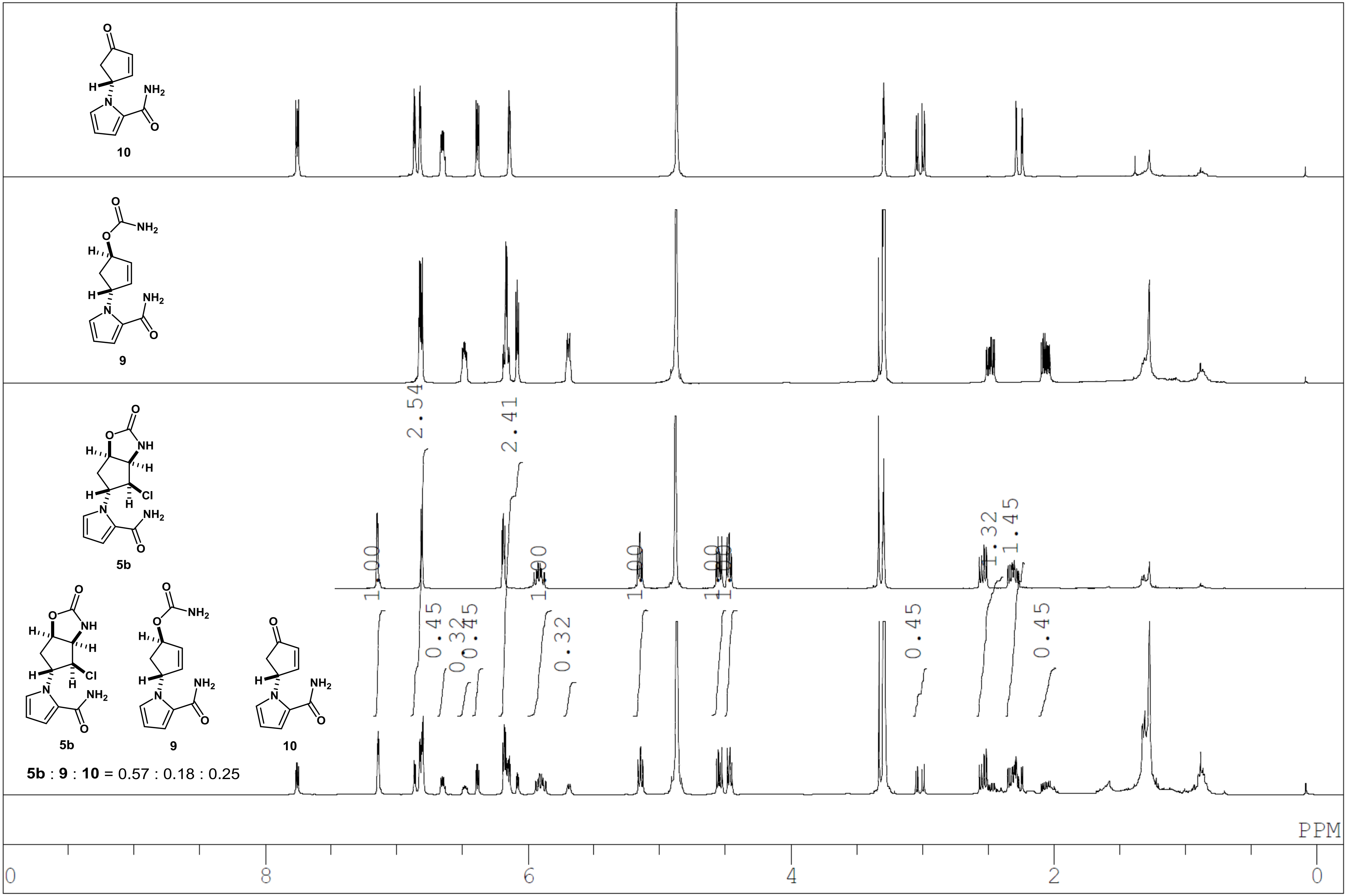

Table 1, entry 6

400MHz, CD_3OD

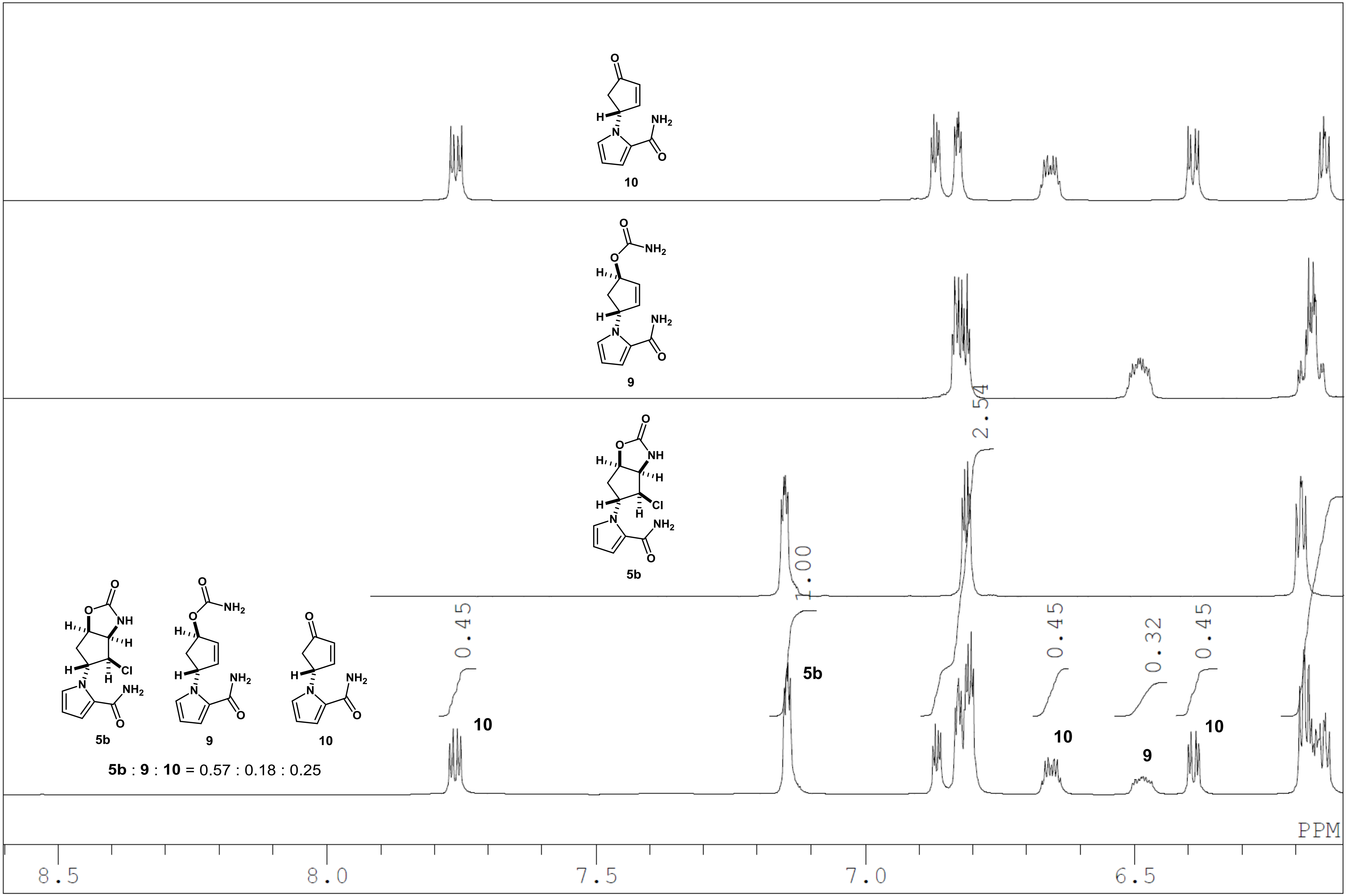

Table 1, entry 7

400MHz, CD_3OD

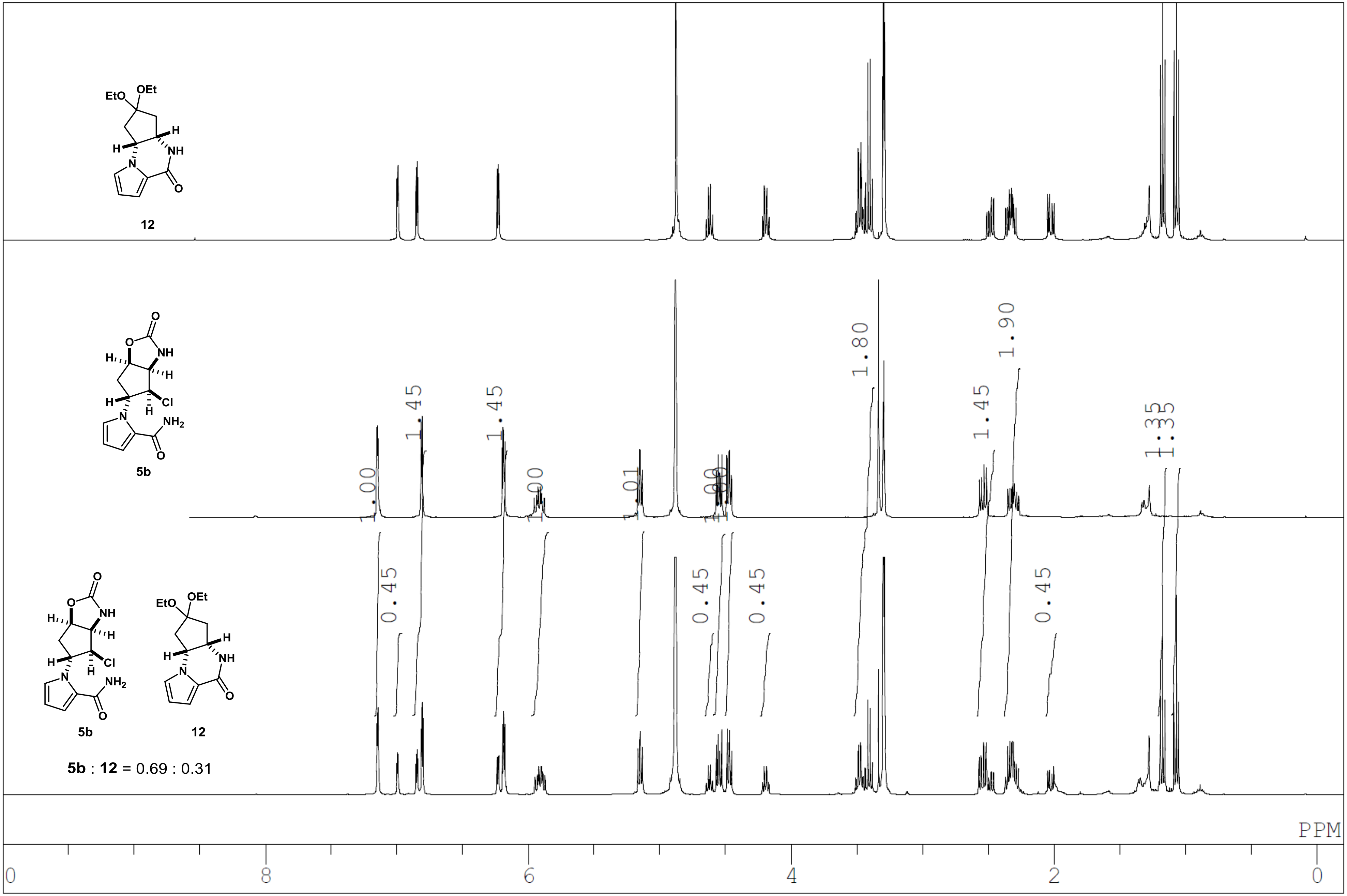

Table 1, entry 7

400MHz, CD_3OD

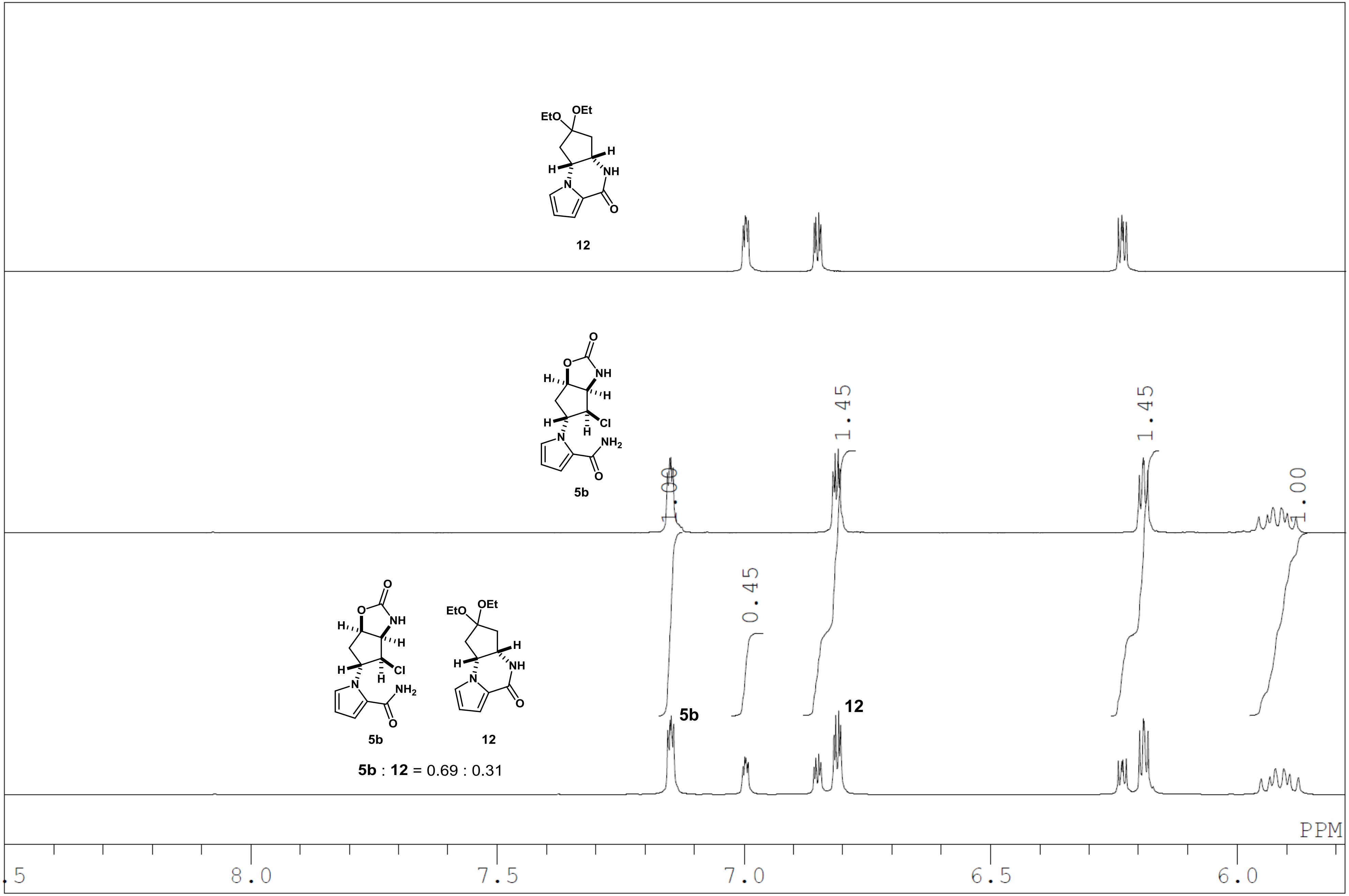

Supplement: File 1 — Experimental procedures, characterization data of new compounds, and 1H/13C NMR spectra. [file Beilstein_J_Org_Chem-09-860-s001.pdf]
